# ER chaperone GRP78/BiP translocates to the nucleus under stress and acts as a transcriptional regulator

**DOI:** 10.1073/pnas.2303448120

**Published:** 2023-07-24

**Authors:** Ze Liu, Guanlin Liu, Dat P. Ha, Justin Wang, Min Xiong, Amy S. Lee

**Affiliations:** ^a^Department of Biochemistry and Molecular Medicine, University of Southern California, Keck School of Medicine, Los Angeles, CA 90033; ^b^Norris Comprehensive Cancer Center, Keck School of Medicine, University of Southern California, Los Angeles, CA 90033; ^c^Department of Molecular Medicine, Scripps Research, La Jolla, CA 92037; ^d^Department of System Biology, Beckman Research Institute, City of Hope, Duarte, CA 91010

**Keywords:** GRP78, nuclear translocation, ER stress, lung cancer, transcriptional regulation

## Abstract

Cancer cells are commonly subjected to endoplasmic reticulum (ER) stress. To gain survival advantage, cancer cells exploit the adaptive aspects of the unfolded protein response such as upregulation of the ER luminal chaperone GRP78. The finding that when overexpressed, GRP78 can escape to other cellular compartments to gain new functions regulating homeostasis and tumorigenesis represents a paradigm shift. Here, toward deciphering the mechanisms whereby GRP78 knockdown suppresses *EGFR* transcription, we find that nuclear GRP78 is prominent in cancer and stressed cells and uncover a nuclear localization signal critical for its translocation and nuclear activity. Furthermore, nuclear GRP78 can regulate expression of genes and pathways, notably those important for cell migration and invasion, by interacting with and inhibiting the activity of the transcriptional repressor ID2. Our study reveals a mechanism for cancer cells to respond to ER stress via transcriptional regulation mediated by nuclear GRP78 to adopt an invasive phenotype.

## Significance

Endoplasmic reticulum (ER) stress-mediated relocalization of ER chaperones to other cellular compartments allows cells to expand their functionality beyond the ER. Our finding that the ER luminal chaperone GRP78/BiP, commonly overexpressed in cancer cells, can translocate to the nucleus represents a paradigm shift about its role in regulating homeostasis and tumorigenesis. This study uncovers a molecular mechanism by which cancer cells respond to stress through nuclear translocation of GRP78/BiP, which assumes a role as a transcriptional regulator, allowing cells to adopt an invasive phenotype and impacting other pathways. Our study further suggests that GRP78/BiP inhibitors may offer a therapeutic approach to suppress EGFR in various human lung cancer cells without the limitations of targeting specific mutations.

Molecular chaperones are increasingly recognized as major regulators of cellular homeostasis in health and disease beyond their originally discovered canonical role as protein foldases (1–6). The 78-kDa glucose-regulated protein (GRP78), also referred to as BiP and encoded by the *HSPA5* gene, is a member of the heat shock protein 70 (HSP70) protein family. Unlike the cytosolic HSP members, GRP78 contains a signal sequence that targets it into the endoplasmic reticulum (ER). As a major ER chaperone, GRP78 is well established to play a critical role in folding and processing of nascent membrane-bound or secretory proteins (7, 8). Through its interaction with the transmembrane ER stress sensors, GRP78 further functions as a key regulator of the unfolded protein response (UPR), which is an evolutionarily conserved mechanism to allow cells to adapt to proteotoxic stress, commonly observed in oncogenic, metabolic, and neurological disorders (2, 9–11).

While GRP78 and other ER chaperones were traditionally regarded as luminal ER proteins, the finding that ER stress not only up-regulates the expression of ER chaperones to cope with ER protein quality control but also actively promotes their relocation to other cellular compartments where they assume unexpected regulatory functions beyond the ER represents a paradigm shift (5, 12–15). For example, GRP78 at the cell surface acts as a multifaceted coreceptor regulating a wide range of signaling pathways as well as virus internalization, while other ER chaperones such as GRP94 and calreticulin at the cell surface assume novel immunoregulatory roles (10, 16–25). GRP78 can localize to the mitochondria under ER stress, modulating mitochondria function and homeostasis (26, 27). Interestingly, GRP78 can be observed in the nucleus when overexpressed or induced by ER stress and cross-linked to DNA in irradiated cells; however, the mechanism of translocation of GRP78 from the ER to the nucleus and its function as a nuclear protein remain largely unknown (28–30).

GRP78 is highly induced in a wide range of tumors through intrinsic factors such as altered glucose metabolism of cancer cells and hyperproliferation, compounded by extrinsic factors such as glucose deprivation, hypoxia, and acidosis in the microenvironment of poorly perfused tumors (2, 31–34). Evidence is emerging that GRP78, in addition to being a potent antiapoptotic protein and a major pro-survival component of the UPR, is a regulator of oncogenic drivers such as PI3K, TGF-β, CD44, and KRAS through direct or indirect interactions (17–19, 35). In examining how GRP78 deficiency suppressed pancreatic cancer initiation and progression in the *Pdx1-cre; KRAS^G12D/+^; p53^f/+^* mouse model, we noted that GRP78 knockdown led to a decrease in EGFR expression (36). EGFR is well established to play fundamental roles in cell proliferation, differentiation, and motility (37). Overexpression or hyperactivation of EGFR is strongly associated with poor prognosis in a wide range of cancers, including lung cancer which is a leading cause of cancer mortality worldwide with limited therapeutic options (38). Recently, we reported that GRP78 is critical for mutant *Kras*-driven lung tumorigenesis, in part due to its regulatory function of the UPR (39). However, other potential mechanisms remain to be explored.

In the present study, using lung cancer cell lines and other cell model systems, we find that GRP78 knockdown in lung cancer led to a reduction in *EGFR* mRNA level and surprisingly, the regulation is at the transcriptional level. This raises the important question whether GRP78 itself can regulate gene transcription in the nucleus. Here, using a combination of molecular, biochemical, bioinformatics, and imaging approaches, we identify a nuclear localization signal (NLS) of GRP78 critical for its nuclear translocation and uncover a transcriptional regulatory mechanism linking nuclear GRP78 to Inhibitor of DNA binding 2 (ID2), a basic helix–loop–helix (bHLH) transcriptional factor which functions as a dominant negative inhibitor of E proteins with tumor suppressor properties in lung cancer (40, 41). Our studies find a role of GRP78 as a direct regulator of gene transcription impacting invasion and migration through interacting and negating the inhibitory effects of ID2.

## Results

### The GRP78 Level is Elevated in Lung Cancer and Its Depletion Reduces EGFR Expression.

**Fig. 1.**
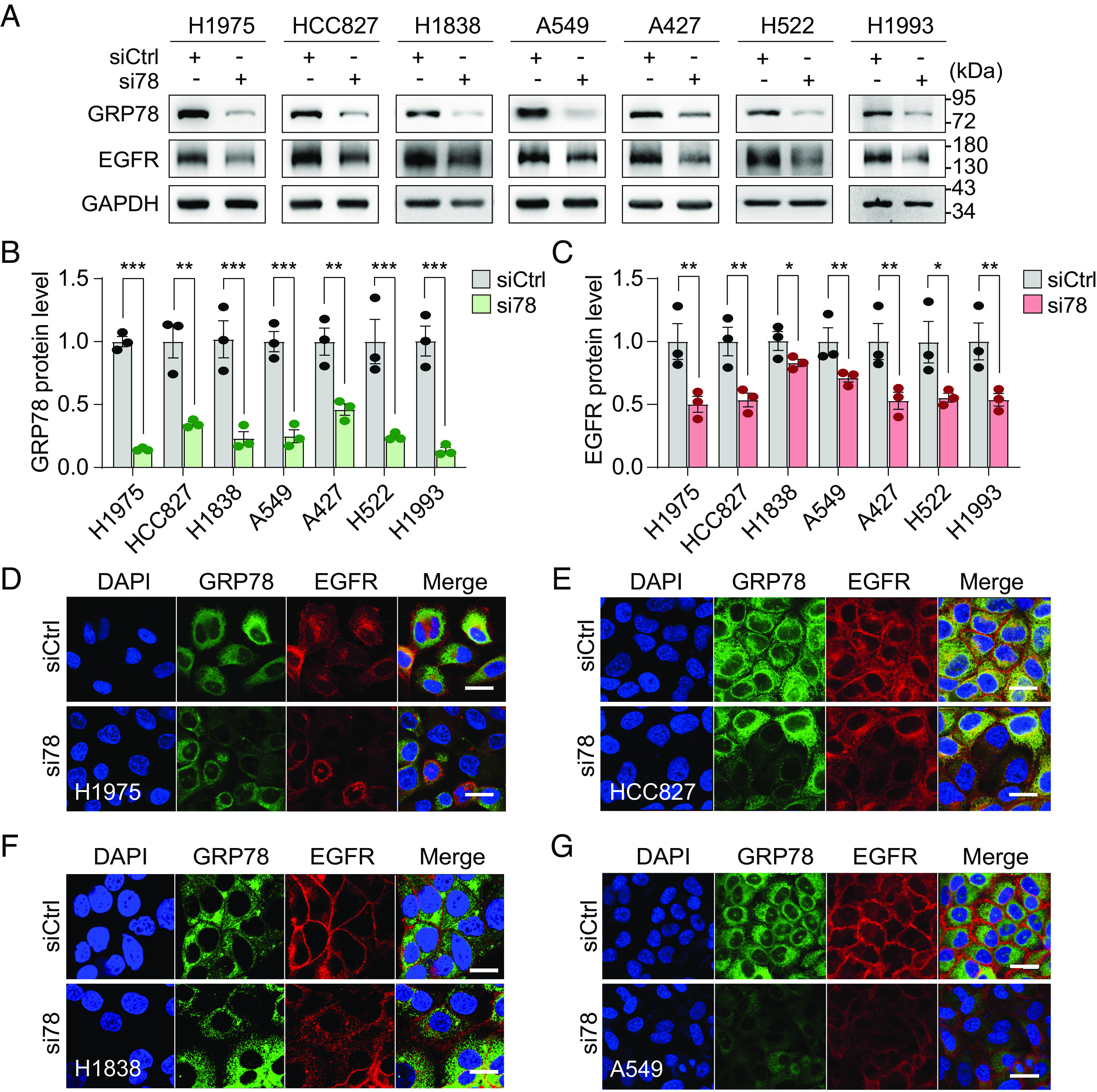
GRP78 knockdown reduces EGFR protein levels in human lung cancer cell lines. (*A*) The indicated human lung adenocarcinoma cell lines were transfected with control siRNA (siCtrl) or siRNA targeting the 3′-UTR of *GRP78* mRNA (si78) for 48 h. Whole cell lysates were subjected to Western blot analysis for GRP78 and EGFR protein levels with GAPDH serving as loading control. (*B* and C) Quantification of the relative protein levels of GRP78 and EGFR, respectively, after normalization against GAPDH levels is shown in the graphs (n = 3). (*D*–*G*) Representative confocal immunofluorescence images of GRP78 (green) and EGFR (red) staining in the indicated cell lines after 48 h treatment with siCtrl or si78. The nuclei were stained by DAPI in blue. (Scale bars, 20 μm.) Data are presented as mean ± SEM. *P ≤ 0.05, **P ≤ 0.01, ***P ≤ 0.001 (Student’s *t* test). See also *SI Appendix*, Fig. S1.

Analysis of the Cancer Genome Atlas (TCGA) database by the GEPIA2 tool (42) showed that *GRP78* mRNA expression in human lung adenocarcinoma (LUAD) is higher than in normal lung tissues and is a poor prognostic marker for survival among LUAD patients (*P* < 0.001) (*SI Appendix*, Fig. S1 *A* and *B*). Considering the importance of EGFR as a therapeutic target in lung cancer, we seek to determine potential links between GRP78 and EGFR. To test this, we knockdown GRP78 by siRNA in a panel of 7 human non-small-cell lung carcinoma cell lines (NSCLCs) bearing different *EGFR* genotypes, including the commonly mutated and amplified *EGFR* alleles (*SI Appendix*, Table S1). The siRNA targets the unique 3′UTR of the human *GRP78* gene (si78) and has been previously shown to efficiently deplete endogenous GRP78 in human cell lines (19) (*SI Appendix*, Fig. S1*C*). Western blot analysis showed that upon GRP78 reduction, the level of EGFR protein was significantly reduced in all seven cell lines (Fig. 1 *A–C*). It is notable that H1838, the cell line least affected by GRP78 depletion, contains highly amplified *EGFR*. These biochemical results were confirmed using confocal immunofluorescence microscopy, showing in cells where GRP78 was depleted by si78 treatment, the level of EGFR expression also decreased correspondingly (Fig. 1 *D–G*). To rule out the off-target effect of the si78 against the 3′UTR, we utilized another siRNA targeting the coding region of the *GRP78* gene to deplete GRP78 and observed that both siRNAs targeting different regions of the *GRP78* gene were able to reduce EGFR protein levels in human lung cancer cell lines (*SI Appendix*, Fig. S1 *C–E*). The same results were observed in the human embryonic kidney HEK293AD cells, which provide a valuable cell model system for biochemical and imaging analyses with high transfection efficiency and strong adhesive properties (*SI Appendix*, Fig. S1*F*).

### GRP78 Knockdown Suppressed EGFR at the Transcriptional Level.

**Fig. 2.**
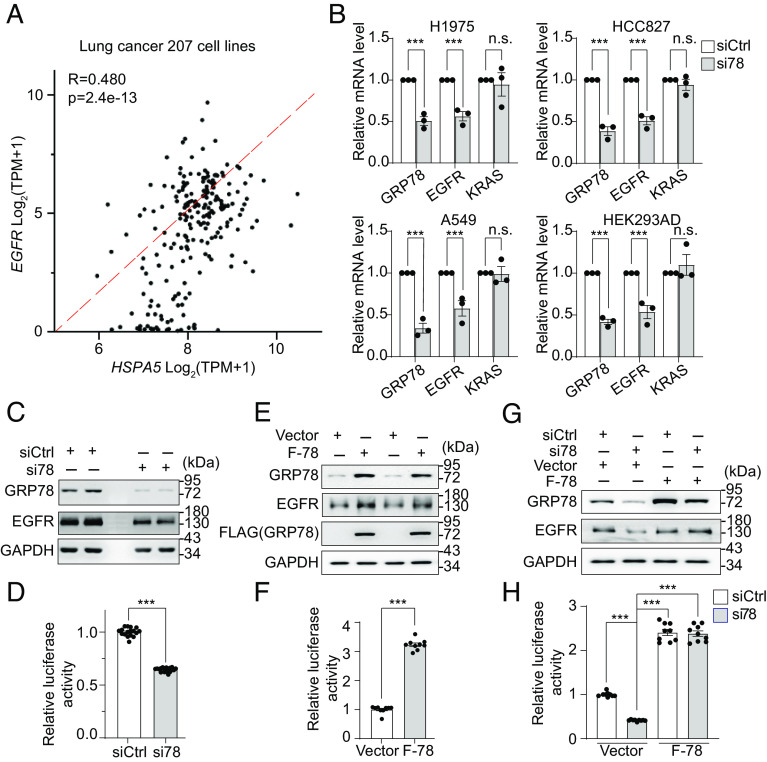
GRP78 regulates EGFR expression at the transcriptional level. (*A*) Correlation between *GRP78* and *EGFR* mRNA expression levels in a panel of 207 lung cancer cell lines from DepMap. (*B*) RT-qPCR analysis of *GRP78, EGFR*, and *KRAS* mRNA levels in the indicated cell lines treated with siCtrl or si78 for 48 h (n = 3). (*C*) HEK293AD cells were transfected with EGFR-Luc reporter gene and siCtrl or si78 for 48 h. GRP78 and EGFR protein levels were analyzed by Western blots with GAPDH serving as loading control, and *EGFR* promoter activity was measured by the dual luciferase assay in (*D*). (*E*) Similar to (*C*) except HEK293AD cells were transfected with the EGFR-Luc reporter gene and empty vector or F-78 expression construct, and *EGFR* promoter activity was measured in (*F*). (*G*) Similar to (*C*) except HEK293AD cells were transfected with EGFR-Luc reporter gene and siCtrl or si78 in combination with empty vector or F-78 expression construct as indicated and *EGFR* promoter activity was measured in (*H*). Data are presented as mean ± SEM. *P ≤ 0.05, **P ≤ 0.01, ***P ≤ 0.001 (Student’s *t* test). See also *SI Appendix*, Fig. S2.

Next, we investigated whether the downregulation of EGFR resulting from GRP78 knockdown is at the transcriptional or translational level. Gene expression correlation analysis of 207 human lung cancer cell lines using the DepMap data explorer (43) revealed that *GRP78* is positively correlated with *EGFR* at the mRNA level (*P* = 2.4e-13, R = 0.48) (Fig. 2*A*), suggesting that GRP78 may regulate EGFR expression at the transcriptional level. To directly test this, we knockdown GRP78 by si78 in human lung cancer lines and HEK293AD cells. The transcript levels of *GRP78* and *EGFR* were measured by RT-qPCR, and we observed that compared to cells treated with control siRNA, cells treated with si78 showed about 50% decrease of *EGFR* mRNA levels (Fig. 2*B*), similar to the decrease of EGFR protein levels in the same cells (Fig. 1 *A* and *C*). In contrast, the mRNA levels of *KRAS* in the same cells were not affected.

To determine whether the reduction of *EGFR* mRNA levels in the si78-treated cells is due to a decrease in *EGFR* mRNA stability, the human lung cancer cells A549 were treated with Actinomycin D, and the mRNA stability was measured in cells treated with either siCtrl or si78. Our results showed that *EGFR* mRNA stability was not affected by GRP78 knockdown (*SI Appendix*, Fig. S2 *A* and *B*). Collectively, these results show that GRP78 knockdown preferentially reduces *EGFR* mRNA levels, and this reduction is likely at the transcriptional level as the mRNA stability of *EGFR* is not affected.

To test this, we created a luciferase reporter construct driven by the human *EGFR* promoter, which contains 1,123 base pairs upstream of the transcription start site reported to be most critical for the *EGFR* promoter activity (44, 45) (*SI Appendix*, Fig. S2 *C*, *Upper*). In the following studies that dissect the mechanism(s) whereby GRP78 regulates transcription of *EGFR*, HEK293AD cells were used as a model system. First, we verified that the EGFR-Luc construct showed robust luciferase activity compared to the vector control (*SI Appendix*, Fig. S2 *C*, *Lower*). Next, using the dual luciferase reporter assay, we showed that knockdown of endogenous GRP78 by si78 (*SI Appendix*, Fig. S2 *D*, *Upper*) suppressed the *EGFR* promoter activity (Fig. 2 *C* and *D*). To blunt si78 action, we utilized a FLAG-tagged expression construct of GRP78 (F-78) which does not contain the si78 targeted 3′UTR region (*SI Appendix*, Fig. S2 *D*, *Lower*). Transfection of F-78 into HEK293AD cells elevated the total GRP78 level in these cells and stimulated *EGFR* promoter activity (Fig. 2 *E* and *F*). Importantly, when F-78 was transfected in combination with si78 into HEK293AD cells, F-78 rescued the suppressive effect of si78 on the *EGFR* promoter activity (Fig. 2 *G* and *H*). Thus, these results provide evidence that GRP78 is an activator of *EGFR* promoter activity.

### GRP78 Localizes to the Nucleus in Human Lung Cancer Cells and Cells under ER Stress.

**Fig. 3.**
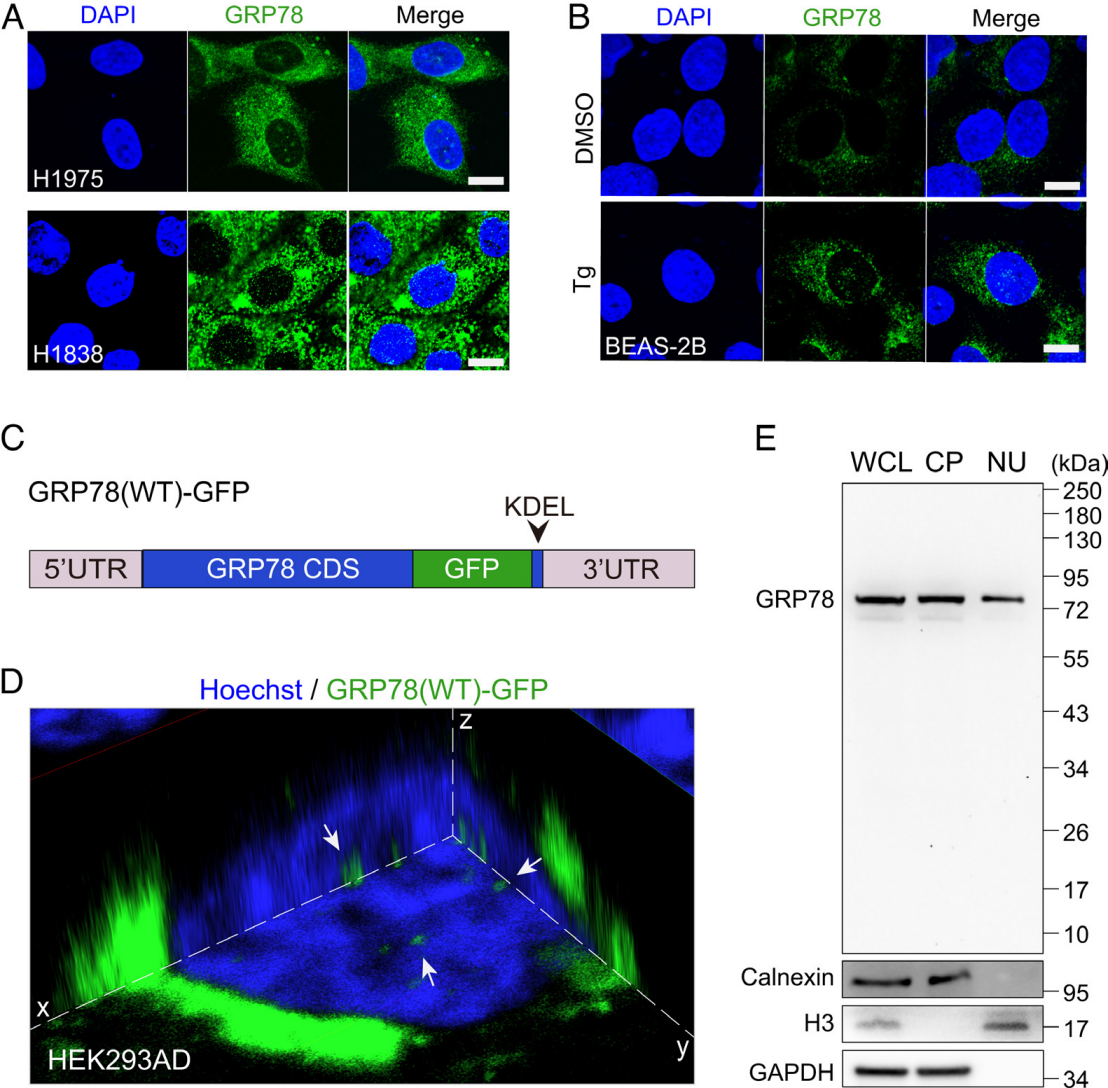
GRP78 translocates to the nucleus in human lung cancer cells and ER-stressed normal lung epithelial cells. (*A*) Representative confocal immunofluorescence images of GRP78 (green) staining in human lung cancer H1975 and H1838 cells. The nuclei were stained by DAPI in blue. (Scale bars, 10 μm.) (*B*) Representative confocal immunofluorescence images of GRP78 (green) staining in normal human lung epithelial BEAS-2B cells treated with DMSO or thapsigargin (Tg, 100 nM) for 16 h. The nuclei were stained by DAPI in blue. (Scale bars, 10 μm.) (*C*) Schematic drawing of GRP78(WT)-GFP, which contains the 5′ and 3′ UTRs flanking the full-length, wild-type GRP78 coding sequence (CDS), with the green fluorescent protein (GFP) tag inserted just prior to the KDEL ER retrieval motif. (*D*) Three-dimensional confocal live cell imaging of HEK293AD cells transfected with the GRP78(WT)-GFP (green) construct for 48 h. The nucleus was stained with Hoechst 33342 (blue). The white arrows indicate GRP78 in the nucleus. (*E*) Western blot of whole cell lysate (WCL), cytoplasmic (CP), and nuclear (NU) fractions of H1838 cells for GRP78 performed with a polyclonal antibody, with calnexin, Histone H3, and GAPDH serving as ER, nuclear, and cytoplasmic markers, respectively. See also *SI Appendix*, Fig. S3.

To address whether GRP78, primarily located in the ER, can translocate to the nucleus under pathophysiological conditions, we examined the localization of GRP78 in human lung cancer cell lines through confocal immunofluorescence microscopy. These include H1975 cells bearing *EGFR* (L858R/T790M) mutation and H1838 cells with amplified wild-type *EGFR*. In both cell lines, GRP78 was readily detectable in the nucleus in addition to localization in the perinuclear region typical of the ER (Fig. 3*A* and *SI Appendix*, Fig. S3*A*). In contrast, GRP78 was not detectable in the nucleus of lung bronchial epithelial cells BEAS-2B under normal culture conditions, however, when the cells were treated with thapsigargin, a well-established ER-stress inducer, GRP78 localization to the nucleus was readily detectable (Fig. 3*B* and *SI Appendix*, Fig, S3*B*).

While the use of anti-GRP78 antibodies to detect GRP78 is useful for studying endogenous GRP78 expression and localization, this requires cell fixation and permeabilization steps which may generate staining artifacts. To exclude these possibilities, we performed live cell confocal imaging of ectopically expressed GRP78-GFP fusion protein in HEK293AD cells. It is noted that the GFP motif was inserted just prior to KDEL ER retention motif of GRP78, such that it does not affect the ER retrieval mechanism of GRP78 (46) (Fig. 3*C*). The Z-stack image of 3D live cell confocal fluorescence microscopy showing a cross-section of the cell confirmed that in addition to the perinuclear staining typical of the ER, multiple spots of GRP78-GFP (green signal) were evidently localized inside the nucleus (blue) denoted by Hoechst 33342 staining (Fig. 3*D*). Additionally, we performed biochemical subcellular fractionation of H1838 cells and subjected the purified fractions to Western blots. We observed primarily full-length, 78-kilodalton GRP78 band in the nuclear fraction, with no major lower molecular weight bands, using either a polyclonal anti-GRP78 antibody (Fig. 3*E*) or a monoclonal antibody (*SI Appendix*, Fig. S3*C*). In the nuclear fraction, there was no detectable contamination from the ER as indicated by the absence of the ER transmembrane protein calnexin (Fig. 3*E* and *SI Appendix*, Fig. S3*C*). As expected, histone H3 protein was present in the nuclear fraction but absent in the cytoplasmic fraction (Fig. 3*E* and *SI Appendix*, Fig. S3*C*). Collectively, these studies establish that GRP78 in human cancer cells, or when it is induced by ER stress or ectopically overexpressed, can translocate to the nucleus, giving rise to potential functional roles for GRP78 in the nucleus.

### Identification of the Nuclear Localization Signal of GRP78 Critical for Its Nuclear Import and Regulation of *EGFR* Promoter Activity.

**Fig. 4.**
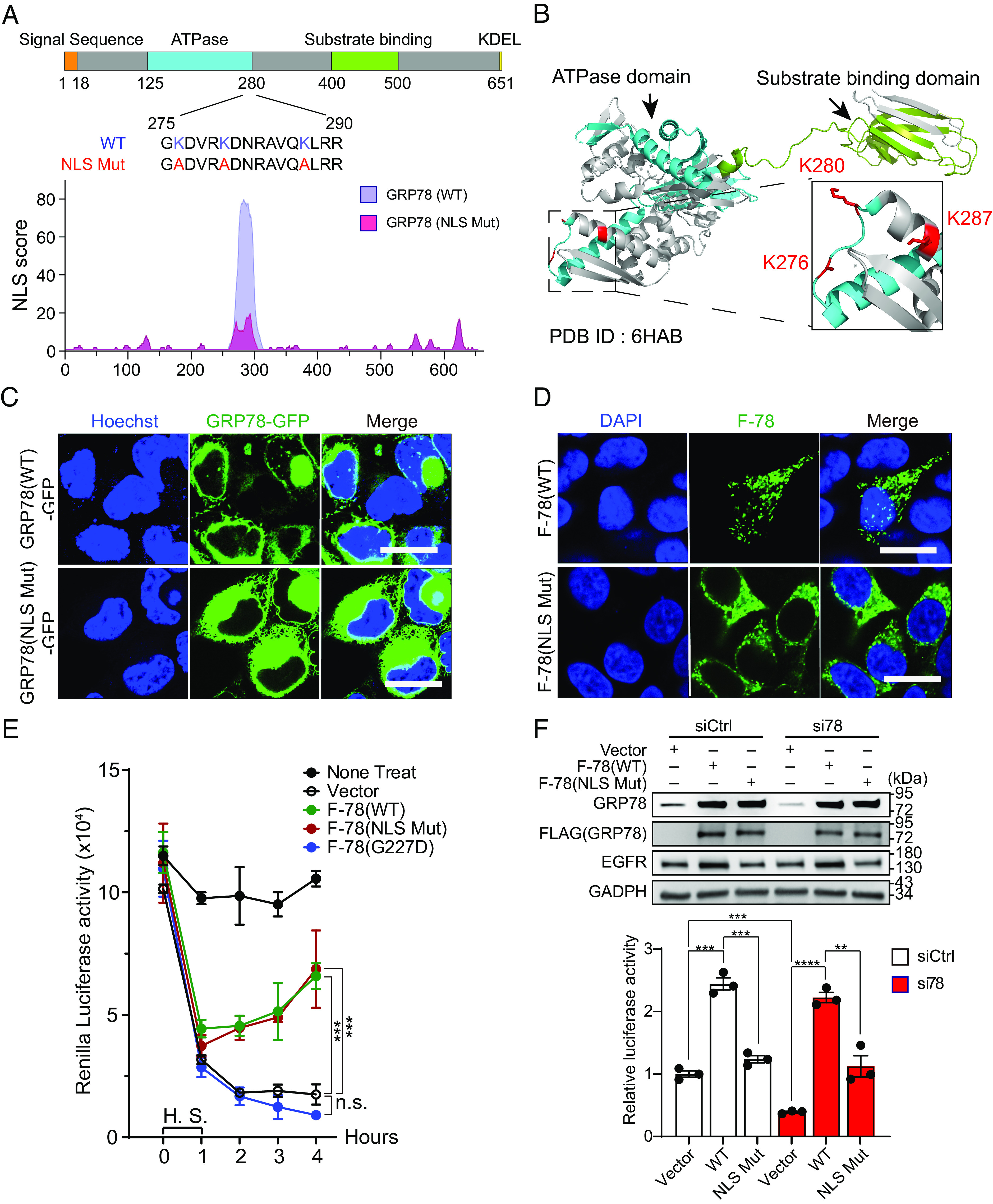
Identification of the Nuclear Localization Signal (NLS) of GRP78 required for its nuclear import. (*A*) Schematic illustration of the functional domains of GRP78 and the putative location of the NLS as predicted by NLStradamus. The plot of the NLS score of the wild-type and mutant NLS is shown below. (*B*) Three-dimensonal crystal structure of GRP78 protein (PDB: 6HAB) showing the ATPase domain in dark cyan color and substrate binding domain in green color. Three lysine residues are labeled in the red and dashed box is magnified view showing these lysine residues near the end of the ATPase domain and their side chain positions. (*C*) Representative confocal live cell images of HEK293AD cells transfected with GRP78(WT)-GFP (*Upper*) or GRP78(NLS Mut)-GFP constructs (*Lower*) for 48 h. The nuclei were stained by Hoechst 33342 in blue. White arrows indicate nuclear GRP78. (Scale bars, 10 μm.) (*D*) Representative confocal immunofluorescence images of HEK293AD cells transfected with F-78(WT) (*Upper*) or F-78(NLS Mut) constructs (*Lower*) for 48 h. The nuclei were stained by DAPI in blue. (Scale bars, 10 μm.) (*E*) HEK293AD cells were transfected with *thymidine kinase* (TK) promoter-driven Renilla luciferase reporter construct and empty vector, or F-78(WT), or F-78(NLS Mut), or F-78(G227D) for 48 h. The cells were subjected to heat shock (H.S.) for 1 h at 42 °C and collected at different timepoints from 0 to 4 h. Renilla luciferase activity in whole cell lysates was measured and graphed (n = 3). (*F*) *Upper*: HEK293AD cells were transfected with either siCtrl or si78 in combination with empty vector or F-78(WT) or F-78(NLS Mut) constructs as indicated for 48 h. The cell lysates were subjected to Western blots for detection of GRP78 and EGFR protein levels with GAPDH serving as loading control. *Lower*: *EGFR* promoter activity was measured by dual luciferase assay (n = 3). Data are presented as mean ± SEM. *P ≤ 0.05, **P ≤ 0.01, ***P ≤ 0.001, ****P ≤ 0.0001 (Student’s *t* test). See also *SI Appendix*, Fig. S4.

To investigate how GRP78 translocates to the nucleus, we queried whether GRP78 contains a NLS, and if so, whether this signal is required for its trafficking to the nucleus. First, we used NLStradamus (47) to search for potential NLS in GRP78. Interestingly, a single cluster composed of 16 amino acids between 275 and 290 of GRP78 was predicted to be a NLS sequence with a relatively high NLS score of 80 (out of 100), with a score of 50 being considered as positive for NLS (Fig. 4*A*). This lysine (K) rich region is highly conserved evolutionarily from *S. cerevisiae* to *H. sapiens* especially in vertebrates by sequence alignment analysis (*SI Appendix*, Fig. S4*A*). Similarly, other chaperones related to GRP78 (HSP70, HSP72, HSP73, HSP90A, HSP90B) also exhibit this highly positively charged region (*SI Appendix*, Fig. S4*B*). Interestingly, the crystal structure of GRP78 (PDB: 6HAB) retrieved from the Research Collaboratory for Structural Bioinformatics Protein Data Bank revealed that the three lysine residues (K276, K280, K287) in the NLS sequence are exposed on the surface of the protein (Fig. 4*B*), which may facilitate their interaction with the nuclear translocation machinery to import GRP78 into the nucleus.

To test the requirement of this putative NLS sequence for GRP78 import to the nucleus, we used GRP78-GFP as template and created a NLS mutant through substituting the three positively charged lysine residues (K276, K280 and K287) with alanine residues, resulting in a drop of the NLS score to 18 which is deemed negative by NLStradamus software (Fig. 4*A*). Through live cell confocal imaging, we established that while GRP78 nuclear localization was evident for GRP78(WT)-GFP, nuclear GRP78 was below the detection limit for GRP78(NLS Mut)-GFP (Fig. 4*C* and *SI Appendix*, Fig. S4*C*). Since the expression of GRP78 tagged with the FLAG epitope allowed the use of highly specific anti-FLAG antibody to detect ectopically expressed GRP78 in cells, we created the F-78(NLS Mut) construct and observed that as in the case of GRP78(NLS Mut)-GFP, F-78(NLS Mut) was defective in nuclear localization compared to F-78(WT) (Fig. 4*D*). Taken together, these results establish that the predicted NLS sequence in GRP78 is important to direct its import to the nucleus.

Since the NLS is located at the C-terminal border of the ATPase domain of GRP78 (Fig. 4*A*), we tested whether the three lysine mutations affect the chaperone function of GRP78. Through protein re-folding assays performed with F-78(WT), F-78(NLS Mut), and the F-78(G227D) bearing an ATPase domain mutation, all ectopically expressed in similar amounts in HEK293AD cells (*SI Appendix*, Fig. S4*D*), we determined that both F-78(WT) and F-78(NLS Mut) were able to refold denatured proteins, whereas the F-78(G227D) was unable to do so (Fig. 4*E*). Thus, while the NLS mutant cannot enter the nucleus, its protein re-folding function remains intact.

To test whether nuclear localization of GRP78 is required for regulation of *EGFR* promoter activity, we first depleted the HEK293AD cells of the wild-type endogenous GRP78 via si78 which targets the 3′UTR of *GRP78*, followed by ectopic expression of either F-78(WT) or F-78(NLS Mut) which did not contain the si78 targeted 3′UTR sequence. Control cells were transfected with siCtrl. As shown by Western blot of the cell lysates, the transfected F-78 vectors produced similarly high amounts of F-78(WT) and F-78(NLS Mut), with the endogenous GRP78 efficiently knockdown by si78 treatment (Fig. 4 *F*, *Upper*). Next, we performed dual luciferase *EGFR* promoter reporter assay to test the *EGFR* promoter activity in these cells, with the pcDNA3 vector–transfected cells serving as the negative control. Our results demonstrated that knockdown of GRP78 by si78 reduced the luciferase activity as expected, and upon overexpression of F-78(WT), robust luciferase activity was restored, in contrast to the minimal increase observed for F-78(NLS Mut) (Fig. 4 *F*, *Lower*). In agreement, overexpression of F-78(WT) but not F-78(NLS Mut) increased endogenous EGFR protein (Fig. 4 *F*, *Upper*) and mRNA levels (*SI Appendix*, Fig. S4 *E* and *F*). Collectively, these results suggest that the transcriptional activation of the *EGFR* promoter by GRP78 requires its nuclear localization.

### Nuclear GRP78 Regulates *EGFR* Transcription through Interaction with ID2.

**Fig. 5.**
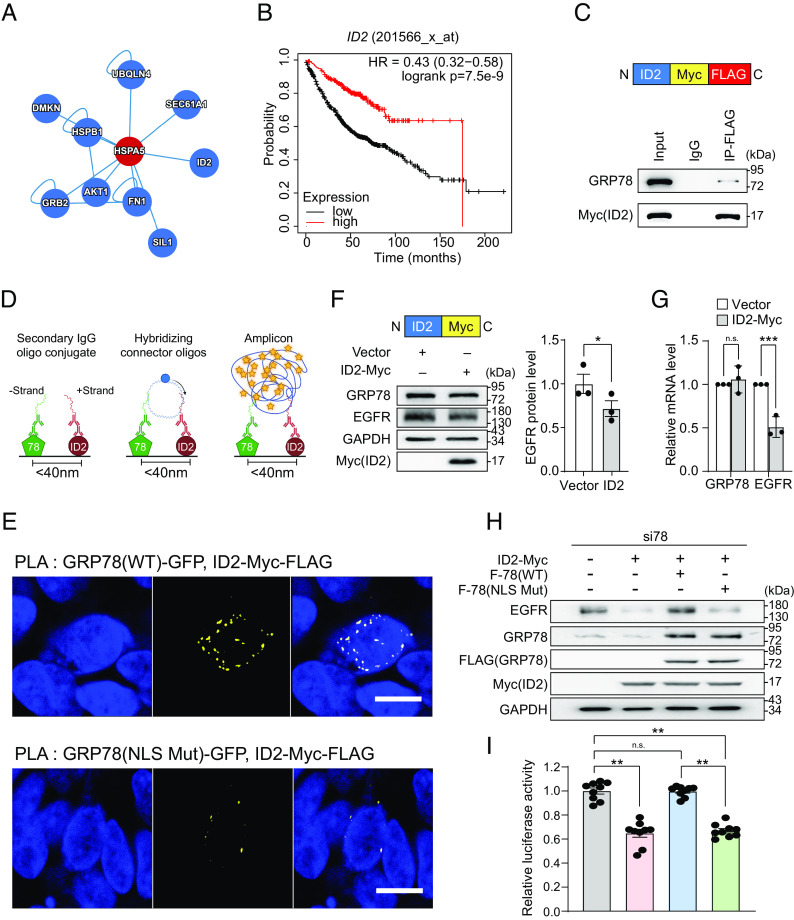
Nuclear GRP78 regulates *EGFR* transcription through interaction with ID2. (*A*) Interaction analysis between GRP78 (HSPA5) and ID2 proteins from the human reference protein interactome mapping project (HuRI). (*B*) Kaplan–Meier survival analysis of LUAD patients with high and low ID2 expression (n = 719) by KM Plotter. (*C*) *Upper*: schematic drawing of ID2-Myc-FLAG. *Lower*: HEK293AD cells were transfected with the ID2-Myc-FLAG and co-immunoprecipitation assays were performed using IgG or anti-FLAG antibodies. The immunoprecipitated proteins were probed for GRP78 and Myc(ID2). (*D*) Schematic diagram of the proximity ligation assay (PLA). (*E*) Representative confocal fluorescence images of PLA between GRP78(WT)-GFP or GRP78(NLS Mut)-GFP and ID2-Myc-FLAG, using antibodies against GFP and FLAG. DAPI (blue) represents nuclei staining, and yellow indicates colocalization. (Scale bars, 10 μm.) (*F*) *Upper*: Schematic diagram of ID2-Myc. *Lower*: HEK293AD cells transfected with ID2-Myc expression construct for 48 h and whole cell lysate was subjected to Western blot for GRP78, EGFR and ID2-Myc proteins with GAPDH serving as loading control. Quantitation of the relative EGFR protein levels normalized to GAPDH was graphed on the right (n = 3). (*G*) Same as in (*F*) except *GRP78* and *EGFR* mRNA levels were measured by RT-qPCR and quantitation of their relative levels normalized to *β-actin* was graphed (n = 3). (*H*) H1975 cells were transfected with EGFR-Luc reporter gene and si78 in combination with empty vector or ID2-Myc or ID2-Myc+F-78(WT) or ID2-Myc+F-78(NLS Mut) as indicated for 48 h. Whole cell lysate was subjected to Western blot for GRP78, EGFR and ID2-Myc proteins with GAPDH serving as loading control. (*I*) Same as in (*H*) except *EGFR* promoter activity was measured by dual luciferase assay. Data are presented as mean ± SEM. *P ≤ 0.05, **P ≤ 0.01, ***P ≤ 0.001, n.s. denotes not significant (Student’s *t* test). See also *SI Appendix*, Fig. S5.

To further investigate the mechanisms through which GRP78 regulates *EGFR* transcription, we queried the human reference protein interactome mapping database (48) to explore potential interacting partners of GRP78. Among the candidates, we found that a nuclear protein ID2 (Fig. 5*A*), which has transcriptional repressor activity, can bind to GRP78 using co-immunoprecipitation (co-IP) mass spectrometry and yeast two-hybrid technologies (49, 50). One mechanism whereby ID2 inhibits gene transcription is through binding and sequestration of E-Box binding proteins. Kaplan–Meier survival analysis further showed that low ID2 expression is a poor prognostic marker for survival among LUAD patients (*P* < 0.001) (Fig. 5*B*).

To validate binding between GRP78 and ID2, we performed the co-IP assay in HEK293AD cells transfected with epitope-tagged ID2 expression construct (ID2-Myc-FLAG) and IP with anti-FLAG antibody. The result showed that ID2-Myc-FLAG can pull down endogenous GRP78 protein, indicating that these two proteins can form a complex (Fig. 5*C*). Similar results were obtained when we overexpressed both ID2-Myc-FLAG and F-78(WT) and IP with anti-Myc antibody (*SI Appendix*, Fig. S5*A*). Furthermore, we utilized the proximity ligation assay (PLA), which reveals protein–protein interactions at distances <40 nm, to assess the close proximity between GRP78 and ID2 (Fig. 5*D*). HEK293AD cells were transfected with GRP78(WT)-GFP or GRP78(NLS Mut)-GFP in combination with ID2-Myc-FLAG and subjected to PLA. Confocal fluorescence analysis revealed that GRP78(WT)-GFP showed extensive interactions with ID2 (depicted in yellow signals) within the nucleus stained by DAPI. In contrast, in cells transfected with GRP78(NLS Mut)-GFP, the yellow signal in the nuclear region was dramatically diminished (Fig. 5*E*). Collectively, these results provide direct evidence that GRP78 can be in close proximity with ID2 in the nucleus and this interaction is dependent on the NLS of GRP78.

To demonstrate the functional importance of ID2 in GRP78 regulation of *EGFR* transcription, we overexpressed ID2 in HEK293AD cells and observed that its elevated protein amount suppressed endogenous EGFR expression at both mRNA and protein levels (Fig. 5 *F* and *G*). Furthermore, ID2-inhibition of endogenous EGFR protein level can be reversed by F-78(WT) but not F-78(NLS Mut) in H1975 human lung cancer cells (Fig. 5*H*) and HEK293AD cells (*SI Appendix*, Fig. S5*B*). Additionally, *EGFR* promoter activity was suppressed by ID2 and rescued by F-78(WT) but not F-78(NLS Mut) in H1975 cells (Fig. 5*I*). Taken together, our data establish that GRP78 regulates *EGFR* transcription through blocking the suppressive activity of ID2, and this effect is dependent on the NLS of GRP78.

### Nuclear GRP78 Regulates Transcription of Genes Important for Invasion and Migration in Human Lung Cancer Cells.

**Fig. 6.**
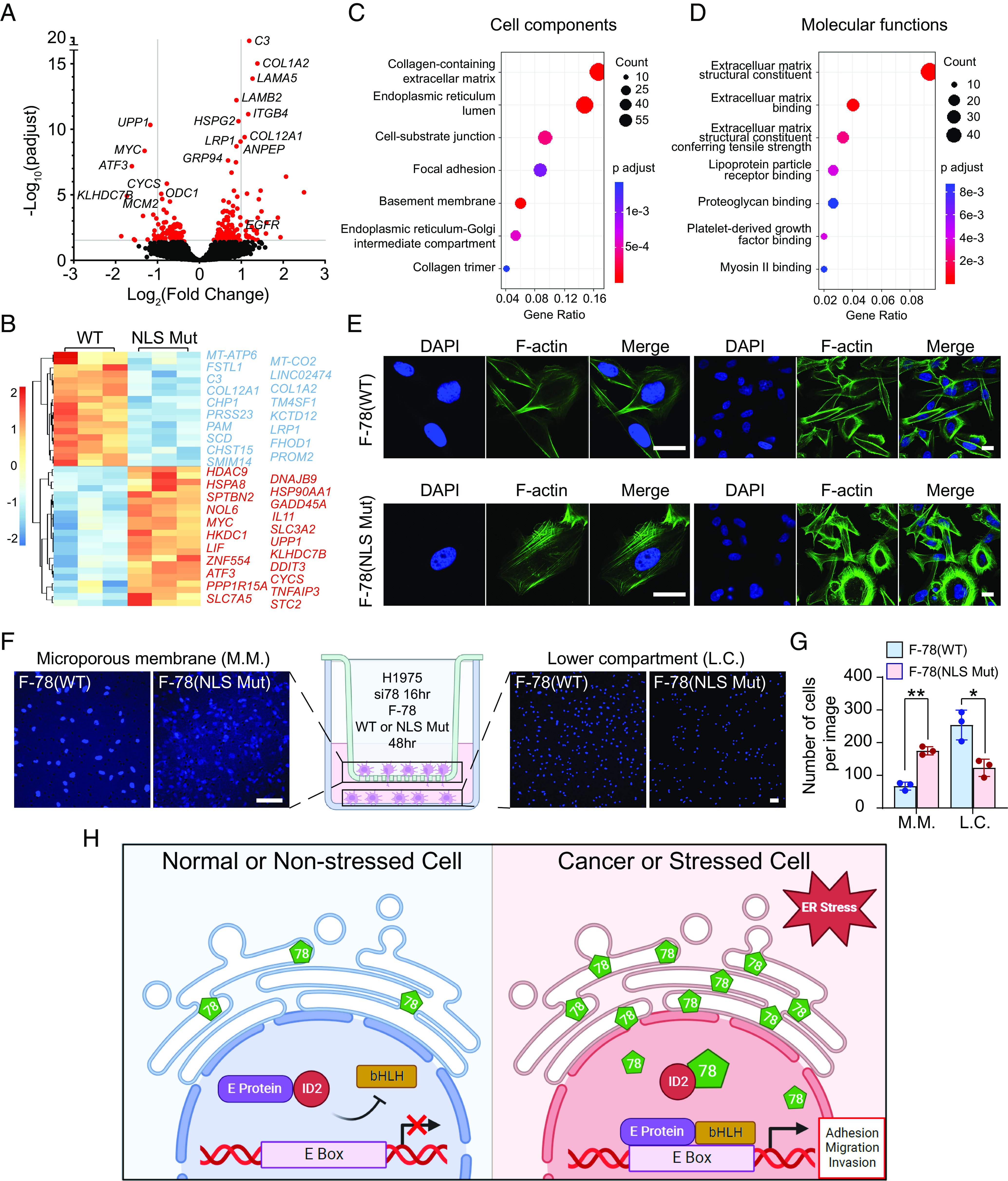
Nuclear GRP78 regulates the expression of genes important for cell migration and invasion in human lung cancer cells. (*A*) Volcano plot for differentially expressed genes in H1975 cells expressing F-78(WT) vs. F-78(NLS Mut). (*B*) Heatmap and clustering of differentially expressed genes from (*A*). (*C* and *D*) Enrichment of cell components and molecular functions of up-regulated genes in F-78(WT) vs F-78(NLS Mut) using ClusterProfiler. (*E*) Representative confocal fluorescence images of H1975 cells transfected with F-78(WT) (*Upper*) or F-78(NLS Mut) constructs (*Lower*) for 48 h and stained for F-actin with phalloidin (green). DAPI (blue) represents nuclei staining. (Scale bars, 10 μm.) (*F*) Representative confocal fluorescence images of DAPI-stained cells remaining on the microporous membrane (M.M.) on the left and cells migrated to the lower compartment (L.C.) on the right in transwell migration assay of H1975 cells transfected with si78 16 h, followed by transfection with F-78(WT) or F-78(NLS Mut) constructs for 48 h. (*G*) Quantification of the number of cells remaining on the M.M. and cells migrated to the L.C. in (*F*). Data are presented as mean ± SEM. *P ≤ 0.05, **P ≤ 0.01, ***P ≤ 0.001 (Student’s *t* test). (*H*) Proposed model for nuclear GRP78-mediated transcriptional regulation via interaction with ID2 under ER stress. In nonstressed cell (*Left*), ID2 binds to E protein and prevents its interaction with bHLH transcription factor. In cancer or stressed cells (*Right*), GRP78 is up-regulated and translocates to the nucleus where it binds and sequesters ID2, relieving the inhibitory effects on transcription leading to activation of genes important for migration and invasion. See also *SI Appendix*, Figs. S6 and S7.

Our finding that nuclear GRP78 can regulate transcription via sequestration of ID2 from its downstream promoters raises the important question of whether nuclear GRP78 could regulate specific gene sets and pathways beyond *EGFR*. To explore this, we performed RNA-Seq analysis to determine the ability of nuclear GRP78 to influence gene expression in the human H1975 lung cancer cell line. Since endogenous GRP78 is already present in the nucleus (Fig. 3*A*), we first depleted the endogenous expression of WT GRP78 by siRNA knockdown (3′UTR) for 24 h. Then, we reconstituted GRP78 expression by transfection of either F-78(WT) or F-78(NLS Mut) with pcDNA3 serving as empty vector control for 48 h. We purified total RNA and performed RNA-Seq analysis to evaluate the effects of nuclear GRP78 on gene expression. We found that cells overexpressing F-78(WT) significantly up-regulated 156 genes and down-regulated 138 genes compared to F-78(NLS Mut) (Fig. 6*A*).

Importantly, pathway analysis revealed that genes involved in cellular responses to stress or regulation of cellular response to stress categories are down-regulated in F-78(WT) or F-78(NLS Mut)-overexpressing cells compared to cells transfected with empty vector control (*SI Appendix*, Fig. S6 *A* and *B*). These results suggest that both WT and NLS Mut GRP78 can perform their traditional chaperone and UPR regulatory functions which are not affected by the status of the NLS. Since we demonstrated that nuclear GRP78 may exert its effects by binding to the transcriptional repressor ID2, which is an E-Box binding protein, we analyzed the promoter regions of the up-regulated genes in F-78(WT) compared to F-78(NLS Mut) and found many E-Box sequences as expected (*SI Appendix*, Fig. S6*C*). This observation supports our hypothesis that GRP78 up-regulates these genes by binding to ID2 and relieving its suppressive effects on the transcriptional activation of these genes. Interestingly, RNA-Seq analysis of ID2 knockdown or knockout has recently been performed in lung adenocarcinoma and sarcoma cells (41, 51). Utilizing these data, we found substantial overlap between the genes up-regulated in cells overexpressing F-78(WT) compared to F-78(NLS Mut) and cells depleted of ID2 (Table 1). To further validate our RNA-Seq results, we utilized a different human lung cancer cell line H1838 and examined the expression level of *COL1A2*, *LRP1*, and *HSP90B1*, three representative genes up-regulated in our RNA-Seq analysis. We found that the expression of all three genes was significantly increased in H1838 cells overexpressing F-78(WT) compared to F-78(NLS Mut) (*SI Appendix*, Fig. S6*D*). Since ER stress further increased nuclear localization of GRP78, we examined the effect of ER stress on the expression of nuclear GRP78 target genes identified in the RNA-Seq analysis such as *COL1A2*, *LRP1*, *HSP90B1*, and *EGFR*. As expected, we observed significant elevation in the mRNA levels of *GRP78* and these genes in normal human lung epithelial cells BEAS-2B under Tg-induced ER stress (*SI Appendix*, Fig. S6*E*).

**Table 1.** Overlap of up-regulated genes in H1975 human lung adenocarcinoma cells expressing F-78(WT) compared to F-78(NLS Mut) identified in this study with gene sets from three other analyses with ID2 knockdown or knockout in human lung adenocarcinoma or Ewing sarcoma cells

Cancer type	Cell line	Method	Overlap	*P*-value	Reference
Lung adenocarcinoma	CL1-0/shID2-528	shRNA knockdown	53 out of 156	1.98e-8	(41)
Ewing sarcoma	TC71-ID2-KO1	CRISPR/Cas9 knockout	71 out of 156	2.16e-18	(51)
Ewing sarcoma	TC71-ID2-KO2	CRISPR/Cas9 knockout	75 out of 156	5.53e-21	(51)

Differential gene expression and clustering analysis revealed that many genes up-regulated in the F-78(WT) group compared to the F-78(NLS Mut) group are involved in cell migration and invasion (Fig. 6*B*). Further analysis of enriched cell components and molecular functions in these up-regulated genes indicated that they are associated with the extracellular matrix or cell motility, (Fig. 6 *C* and *D*) which are both important aspects of migration and adhesion phenotype. Our results are in direct agreement with a recent report that ID2 exerts its tumor suppressor properties in lung cancer through its effects on cancer cell invasion and migration (41). Kaplan–Meier analysis further uncovered that high expression of the most significantly up-regulated genes in F-78(WT) is associated with poor survival among LUAD patients (*SI Appendix*, Fig. S7 *A*–*I*), which is consistent with the pro-tumorigenesis role of GRP78.

To validate the ability of nuclear GRP78 to promote cell migration and motility, we assessed the cytoskeletal organization by staining F-actin bundle with FITC-conjugated phalloidin dye. Confocal fluorescence images of H1975 cells treated with si78 and overexpressing F-78(WT) exhibited an elongated spindle shape with well-developed lamellipodia compared to a round shape with disorganized cytoskeletal structure in cells overexpressing F-78(NLS Mut) (Fig. 6*E*). Additionally, transwell migration assay was performed to examine the effects of nuclear GRP78 on cell migration and invasion. Here, we observed that H1975 cells treated with si78, and overexpressing F-78(WT) can migrate across the microporous membrane to the lower chamber in significantly higher number compared to cells overexpressing F-78(NLS Mut) (Fig. 6 *F* and *G*). Thus, our findings suggest that GRP78 can localize to the nucleus via a NLS and interact with the transcriptional repressor ID2 to regulate the transcription of genes important for migration and invasion (Fig. 6*H*).

## Discussion

GRP78, a major stress-inducible ER chaperone, is up-regulated in a wide range of cancer and associated with aggressive growth, invasive properties, and therapeutic resistance (2, 34, 52–54). Under normal physiological conditions, GRP78 is primarily localized in the ER. Interestingly, upon ER stress, GRP78 can undergo alternative splicing of nuclear pre-mRNA, yielding a cytosolic isoform that regulates PERK signaling and promotes leukemic cell survival (55). Furthermore, the finding that when overexpressed GRP78 can escape from the ER and translocate to other cellular compartments, as well as being secreted via exosomes, to influence cell survival, proliferation, and migration opens frontiers for exploring its trafficking and function beyond the ER (2, 13, 56). While there have been many exciting studies characterizing a wide variety of coreceptors and signaling functions of GRP78 at the cell surface (16, 18–21, 57, 58), with clinical applications in antibody–drug conjugates and CAR T-therapeutics for tumor-specific targeting (59, 60), few studies have described the functions of GRP78 in the nucleus. In this report, we delineated the essential elements for the translocation and activity of nuclear GRP78. Our study uncovered several observations that expand on the unconventional roles of chaperones in health and disease.

First, we found that GRP78 knockdown consistently suppresses EGFR protein expression level in a wide variety of human lung cancer cell lines harboring different *EGFR* mutational and amplification status. This was observed in biochemical analysis as well as in imaging studies where immunofluorescence staining showed cell surface and intracellular EGFR both diminished where upon GRP78 knockdown. EGFR is well known to play diverse roles in tumorigenesis, proliferation, survival, and metastasis in many human malignancies including lung cancer. Wild-type amplification or constitutively active mutant of EGFR constantly transmits signal to downstream pathways to promote cancer initiation and progression. Currently available EGFR therapies primarily target either wild-type or a specific mutant version of this receptor (61). In contrast, our studies showed that targeting GRP78 can suppress EGFR expression irrespective of their mutational or amplification status, thus potentially overcoming such therapeutic limitation in cancer treatment.

Second, upon examination of database for gene expression in 207 human lung cancer cell lines, we detected a statistically significant positive correlation between *GRP78* and *EGFR* transcript levels. Following confirmation that knockdown of GRP78 reduced *EGFR* mRNA levels in various cell lines, we further found that GRP78 regulates *EGFR* promoter activity but not its mRNA stability. In contrast, the mRNA level of another well-known oncogene *KRAS* is not reduced by si78, consistent with our recent finding in other cancer cell lines being tested (35). This result implies that knockdown of GRP78 does not cause widespread global, nonspecific transcriptional shutdown, rather, specific mechanism(s) may be activated by nuclear GRP78 to modulate transcription of specific genes.

Third, in principle, GRP78 as a key chaperone with a wide repertoire of client proteins, can directly or indirectly influence gene transcription in multiple cellular locations. Thus, cell surface GRP78, by mediating various signaling pathways, has been implicated as a transcriptional modulator in cancer (62). Here, we pursue the angle that GRP78 translocates from the ER to the nucleus under pathophysiological conditions to exert its influence on gene transcription. Utilizing confocal microscopy, we established in human lung cancer cell lines harboring mutant or amplified *EGFR* that high level of GRP78 was readily observed in the perinuclear region typical of the ER as well as inside the nucleus. In contrast, in normal human lung epithelial cells which expressed basal level of GRP78, we failed to detect GRP78 in the nucleus under normal culture conditions. However, upon ER stress, a higher level of GRP78 was detected, correlating with the detection of GRP78 in the nucleus. To further confirm that the presence of nuclear GRP78 is not an artifact such as punctae formation due to indentations of the ER through the nucleus, we employed live cell imaging using a GRP78 construct fused with a GFP reporter with the KDEL motif retained at the C-terminus of the fusion protein to preserve the ER-retrieval signal for GRP78. This allowed us to visualize nuclear localization of the GRP78-GFP fusion protein in live cells without the possible confounding factors resulted from staining of fixed, permeabilized cells. Additionally, we performed biochemical subcellular fractionation followed by Western blot analysis and observed full-length GRP78 in the nuclear fraction without major lower molecular size bands. Collectively, these results indicate that an elevated level of GRP78, typically observed in cancer or stressed cells, translocates to the nucleus.

To dissect the mechanism for GRP78 nuclear translocation, we performed a predictive analysis of putative NLS in the GRP78 protein sequence and found that GRP78 contains a strong NLS. This sequence is located near the carboxyl end of the ATPase domain and contains an array of positively charged amino acids such as lysine and arginine. Since the large positively charged amino acids exposed on the surface of proteins are well known to facilitate interaction with nuclear translocation machinery (63), we mutated these positively charged lysine residues to neutral alanine and found that this mutated version of GRP78 failed to translocate to the nucleus. Thus, this provides evidence that GRP78 requires the integrity of its NLS to translocate into the nucleus. Next, we addressed whether the mutations of the charged residues in NLS adjacent to the ATPase domain of GRP78 affects the chaperone function of GRP78. Protein re-folding assays performed with wild-type and NLS mutant showed that the NLS mutations have no effect on the canonical functions of GRP78 as a foldase. Importantly, compared to wild-type GRP78, the NLS mutant failed to rescue *EGFR* transcriptional activity. Thus, while the NLS mutant retains its chaperone function, it cannot relocate to the nucleus and cannot stimulate transcription of the target genes. Regarding the NLS, another point of interest is that lysine and arginine residues are well established to be frequent targets of posttranslational modifications (PMTs) which can shield their positive charges and change the polarity as well as functions and activities of the proteins, it is tempting to speculate that PMTs may contribute to nuclear translocation of GRP78. Furthermore, recent reports that ER luminal proteins can reflux to the cytosol as properly folded entities (5, 64, 65) raise the interesting question on the route(s) whereby GRP78 enters the nucleus, which remains to be determined.

As a first step toward understanding how GRP78 regulates gene transcription, we utilized the human reference protein interactome mapping database to identify nuclear factors that interact with GRP78. This approach led to the finding of ID2 as a binding partner of nuclear GRP78, and here, we validated GRP78 can form complex with ID2 through reciprocal co-IP studies. ID2 is a well-known transcriptional suppressor which prevents binding between the E-protein and the bHLH transcription factor by forming nonfunctional heterodimers with E-protein that are unable to bind DNA (66). The proximity ligation assay further provided in situ evidence that ID2 can be in close proximity to GRP78 in the nucleus. We speculate that GRP78 binding to ID2 will sequester it away from inhibiting E-protein and bHLH interaction, leading to transcriptional activation of the downstream gene targets. Interestingly, TCGA analysis revealed that high ID2 expression correlates with favorable prognosis in human lung cancer patients which is opposite to GRP78. This observation is consistent with our proposed antagonistic relationship between GRP78 and the lung tumor suppressor ID2. Furthermore, it supports the emerging notion that upon ER stress, ER luminal proteins can exit the ER and gain new function as blockers of tumor suppressors, as exemplified by cell surface GRP78 forming a complex with CD109 and blocks TGF-β signaling (18), and cytosolic AGR2 as inhibitor of p53 (5), thus providing a selective advantage to tumor cells.

Importantly, RNA-Seq and bioinformatics analysis revealed pathways controlled by nuclear GRP78. Strikingly, our results showed that the top genes elevated by nuclear GRP78 but not the NLS mutant are involved in cell migration and invasion, and cluster profiling of cell components and molecular functions further indicated that they are associated with the extracellular matrix and adhesion phenotype. Recently, it was reported that ID2 exerts tumor suppressor properties in lung cancer through its effects on cancer cell invasion and migration (41). In that study, besides RNA-Seq analysis, ID2 knockdown promoted lung cancer aggressiveness and led to increase of the cells’ ability to metastasize *in vivo*. On the other hand, the level of GRP78 is increased in metastatic cancer cell lines, lymph node metastasis and human metastatic lesions, and GRP78 knockdown suppresses tumor cell invasion in vitro and suppresses metastatic growth in xenograft and syngeneic tumor models (2). In this report, we validated the ability of nuclear GRP78 to regulate cytoskeletal organization and promote cell migration and invasion. Thus, while cell stress and the UPR have been implicated in cancer cell migration and invasion (67–69), nuclear GRP78 represents a factor contributing to the functional role of GRP78 in metastasis. Nonetheless, since GRP78 is a chaperone which can bind to many client proteins, the key question that follows is whether interaction with ID2 a major mechanism of action of nuclear GRP78. In support of this notion, we found statistically significant and substantial overlap between the up-regulated genes in ID2 depletion and nuclear GRP78 overexpression. This strongly implies that ID2 is a major player in nuclear GRP78 activities and functions, and the consequences are likely to be context dependent as ID2 is reported to play different roles in different cancers. Additionally, there are likely other interacting partners with nuclear GRP78 beyond ID2 and other mechanisms that remain to be explored. In conclusion, our study reveals a potential molecular mechanism of human lung cancer metastasis, mediated by upregulation of GRP78 and its translocation to the nucleus, leading to sequestration of ID2 and activation of genes and pathways impacting migration and invasion. Future studies on this and other functions of nuclear GRP78 warrant vigorous investigations.

## Materials and Methods

Cell culture, plasmids construction, transfection, immunoblot analysis, immunofluorescence, RT-qPCR, RNA stability assay, luciferase reporter assay, live cell imaging, subcellular fractionation, GRP78 protein refolding assay, co-immunoprecipitation, proximity ligation assay, RNA-Seq analysis, bioinformatics analysis, cell invasion transwell assay, and statistical analysis can be found in *SI Appendix*, *Materials and Methods*.

## Supplementary Material

Appendix 01 (PDF)

## Data Availability

The source data for RNA-Seq have been deposited in NCBI Gene Expression Omnibus (GEO) database, https://www.ncbi.nlm.nih.gov/geo/ ( accession number: GSE232661) (70). All other study data are included in the article and/or *SI Appendix*.
