# Supplementary material for: ER chaperone GRP78/BiP translocates to the nucleus under stress and acts as a transcriptional regulator
Source: Proc Natl Acad Sci U S A. 2023 Jul 24;120(31):e2303448120. doi: 10.1073/pnas.2303448120 (PMC10400976; doi:10.1073/pnas.2303448120)
Supplement: Supplementary file 1 — Appendix 01 (PDF) [file pnas.2303448120.sapp.pdf]

Supplemental Information

ER chaperone GRP78/BiP translocates to the nucleus under stress and acts a transcriptional regulator

Ze Liu, Guanlin Liu, Dat P. Ha, Justin Wang, Min Xiong, Amy S. Lee

Supplemental Materials and Methods

Supplemental Figures S1-S7 and Legends

Supplemental Table S1 and Legend

Supplemental Materials and Methods

Cell lines and culture conditions

Human non-small cell lung cancer cell lines H1975, HCC827, H1838, A549, A427, H522, and H1993 were kindly provided by Prof. Steven M. Dubinett (University of California, Los Angeles). Human embryonic kidney cells HEK293AD was provided by Prof. Xiang-Lei Yang (Scripps Research). Human normal lung bronchial epithelial cell line BEAS-2B was provided by Dr. Ite Offringa (University of Southern California). H1975, HCC827, H1838, A427, H522, and H1993 cells were cultivated in RPMI-1640 Medium (Fisher Scientific) supplemented with 10% fetal bovine serum (FBS; GeminiBio, West Sacramento, CA) and 1% penicillin/streptomycin (pen/strep; Corning Inc., Glendale, AZ). HEK293AD cells were cultivated in Dulbecco's Modified Eagle Medium (DMEM) (Fisher Scientific) supplemented with 10% FBS and 1% pen/strep. A549 cells were cultured in F-12K medium containing 10% FBS and 1% pen/strep. Cells were grown at 37°C in a humidified atmosphere of 5% CO_2 and 95% air.

Plasmids

For construction of the luciferase reporter plasmid, 1136 bp of the *EGFR* promoter DNA (-1123 to +13 of *EGFR* gene transcription start site) was generated by PCR amplification from HEK293T genomic DNA and subcloned into the KpnI/XhoI sites of pGL4.17 (luc2/Neo) (Promega) to yield human *EGFR* promoter luciferase construct. The construction of the wild-type and G227D mutant FLAG-tagged GRP78 (F-78) constructs have been described previously[1]. The GRP78-GFP construct was kindly provided by Dr. Erik Snapp (Janelia Research Campus, Ashburn, VA). To generate the nuclear localization signal mutant clones, the F-78 or GRP78-GFP construct was used as template for PCR and the three lysine residues were mutated into alanine (K276A, K280A, K287A) sequentially by site directed mutagenesis. After PCR, DpnI (NEB, R0176L) was

used to cleave the methylated DNA template at 37°C for one hour. The ID2-Myc-FLAG construct (RC205324) was purchased from ORIGENE (Rockville, MD). The ID2-Myc construct was created using the ID2-Myc-FLAG as template for PCR and a nonsense mutation was created before FLAG-tag by site directed mutagenesis. All plasmid sequences were verified by sanger sequencing by Sangon Biotech (Shanghai, China) or Genewiz (South Plainfield, NJ). Sequences of the cloning primers were as follows:

EGFR-promoter:

Forward: 5'-TAGGGTACCTTGCTCCCCTTCAGAGAC-3'

Reverse: 5'-TAGCTCGAGCTGCCCGGACGTCTAG-3'

F-78-K276A:

Forward: 5'-GACGGGCGCAGATGTCAGG-3'

Reverse: 5'-CTATTGTCTGCCCTGACATCTGCG-3'

F-78-K280A:

Forward: 5'- GATGTCAGGGCAGACAATAGAG-3'

Reverse: 5'- CAGCTCTATTGTCTGCCCTG-3'

F-78-K287A:

Forward: 5'-GAGCTGTGCAGGCACTCC-3'

Reverse: 5'-GCCGGAGTGCCTGCAC-3'

GRP78-GFP-K276A:

Forward: 5'-CTGTACAAAAAGAAAACTGGGGCAGAC-3'

Reverse: 5'-GTTGTCTGCTCTAACGTCTGCC-3'

GRP78-GFP-K280A:

Forward: 5'-GAAAGACGTTAGAGCAGACAAC-3'

Reverse: 5'-GCTCTGTTGTCTGCTCTAAC-3'

GRP78-GFP-K287A:

Forward: 5'-CAGAGCTGTGCAGGCACTTC-3'

Reverse: 5'-CCTCACGACGAAGTGCCTG-3'

ID2-Myc:

Forward: 5'-GCAAATGATATCCTGGATTAAAAG-3'

Reverse: 5'-CTTATCGTCGTCATCCTTTTAATC-3'

Transfection of plasmids and siRNAs

Transfection of plasmids was performed with Lipofectamine 3000 (ThermoFisher Scientific, Waltham, MA, Cat# L3000015) and transfection of siRNAs was performed with Lipofectamine RNAiMAX (ThermoFisher Scientific, Cat#136778075) following the manufacturer's instructions. The custom siRNAs were purchased from GE Healthcare Dharmacon Inc. (Chicago, IL). The sequence of siRNAs are as follows:

si78(CDS): 5'-GGAGCGCAUUGAUACUAGA-3'

si78(3'UTR): 5'-CUUAAGUCUCGAAUGUAAU-3'

siCtrl: 5'-GAGAUCGUAUAGCAACGGU -3'

Immunoblot analysis

Cells were lysed in cold lysis buffer (0.5% NP-40, 50 mM Tris-HCl, pH 7.5, 100 mM NaCl, 0.1 mM EDTA, 10% glycerol and 1 mM dithiothreitol) supplemented with 1× protease/phosphatase inhibitor cocktail and the lysate was subjected to 8% or 10% SDS-PAGE. Proteins were transferred onto nitrocellulose membranes at 4°C at 35V overnight. For protein detection, the following primary antibodies were used: mouse monoclonal anti-BiP/GRP78 (1:1000, BD Biosciences, #610978), sheep polyclonal anti-GRP78 (1:1000, Affinity Biologicals, Inc., #SAGRP78-AP), rabbit anti-Calnexin (1:1000, Enzo Life Sciences, ADI-SPA-860), rabbit anti-Histone H3 (1:1000, Santa Cruz Biotechnology, sc-10809), rabbit anti-EGFR (1:1000, Cell signaling technology, #4267), mouse anti-GAPDH (1:5000, Santa Cruz Biotechnology, sc-32233), mouse anti-FLAG M2 mouse antibody (1:1000, Sigma-Aldrich, F1804), mouse anti-Myc-tag (1:1000, Santa Cruz Biotechnology, sc-40). The secondary antibodies used in this study are as follows: m-IgGκ BP-HRP (1:5000, Santa Cruz Biotechnology, sc-516102), mouse anti-rabbit (1:5000, Santa Cruz Biotechnology, sc-2357). Protein bands were visualized by ChemiDoc XRS+ imager (Bio-Rad Laboratories) and quantified by ImageLab Software Version 4.0.1 build 6 (Bio-Rad Laboratories).

Immunofluorescence

Cells were seeded on Millicell EZ SLIDE (MilliporeSigma, PEZGS0816) and fixed with 4% paraformaldehyde and permeabilized in 0.25% Triton X-100 for 10 min at room temperature. After incubation with blocking buffer (5% BSA, 0.1% Tween-20, PBS) for 1 hr, cells were incubated at 4°C overnight with primary antibodies diluted in PBS in a humidified chamber at 4°C. The primary antibodies used were as follows: mouse anti-GRP78 (1:500, MAb159, Gift from Dr. Parkash Gill, USC), rabbit anti-EGFR (1:500, Cell signaling technology, CST #4267), mouse anti-FLAG M2

(1:500, Sigma-Aldrich, F1804). Cells were washed three times with PBS and were incubated with Alexa Fluor-conjugated secondary antibodies for 30 min at room temperature, followed by three more washes with PBS. The secondary antibodies used in this study were Alexa Fluor 488 goat anti-mouse antibody (1:500, Thermo-Fisher Scientific, #A-11001) and Alexa Fluor 594 goat anti-rabbit antibody (1:500, Thermo-Fisher Scientific #A-11012). Cells were then stained with 0.1 μg/ml 4,6-diamidino-2-phenylindole (DAPI) and mounted with VECTASHIELD Antifade Mounting Medium with DAPI (Vector Laboratories, Inc., #H1200). Cell images were acquired with Leica SP8 LIGHTNING Confocal Microscope using a 63× oil objective.

RT-qPCR

For RNA extraction, cells were washed three times with PBS and lysed directly with 1 ml of TRI Reagent (MilliporeSigma) following the manufacturer's instructions. Two micrograms of purified RNA were reverse transcribed using qScript XLT cDNA SuperMix (Quantabio) following the manufacturer's instructions. One microliter of the resulting cDNA was used in real-time PCR (35 cycles: 30 s at 95°C, 30 s at 58°C, 45 s at 72°C). Samples were tested in triplicate using the SYBR Green Super mix (BioRad) on the Strata gene MX3000P Real-Time QPCR System (Agilent). The primers used for each gene are as follows:

EGFR

Forward: 5'-AACACCCTGGTCTGGAAGTACG-3'

Reverse: 5'-TCGTTGGACAGCCTTCAAGACC-3'

GRP78

Forward: 5'-GGTGAAAGACCCCTGACAAA-3'

Reverse: 5'-GTCAGGCGATTCTGGTCATT-3'

KRAS

Forward: 5’-GACTGAATATAAACTTGTGGTAGTTGGA-3’

Reverse: 5’-CATATTCGTCCACAAAATGATTCTGA-3’

COL1A2

Forward: 5’-CTGCTGGAAGTCGTGGTGAT-3’

Reverse: 5’-ACGAAGCCCTTCTTTCCCAG-3’

LRP1

Forward: 5’-CTGGCGAACAAACACACTGG-3’

Reverse: 5’-CACGGTCCGGTTGTAGTTGA-3’

HSP90B1

Forward: 5’-GCCAGTTTGGTGTCGGTTTC-3’

Reverse: 5’-GGGTAATTGTCGTTCCCCGT-3’

β-actin

Forward: 5’-TACCACAGGCATTGTGATGG-3′,

Reverse: 5’-TTTGATGTCACGCACGATTT-3′.

RNA stability assay

A549 cells were transfected with either siCtrl or si78(3’UTR) for 24 hr. The cells were then treated with 5 mg/ml of Actinomycin D (MedChemExpress LLC, Monmouth Junction, NJ). Cells were

collected at 0-, 1-, 2-, 6-, 12- and 24-hr intervals and total RNA was extracted using TRI Reagent. Reverse transcription and real-time PCR analysis were performed as described above.

Luciferase reporter assay

HEK293AD cells were seeded on 6-well plates and transfected as described. After 24 hr, cell extracts were prepared using Dual-Luciferase® Reporter Assay System (Promega) according to manufacturer's instructions. Luciferase activity was detected by a luminometer (BMG LabTech FLUOstar OPTIMA) when Luciferase Assay Buffer (Promega) was added. All experiments were performed in triplicate.

Live cell imaging

Wildtype or NLS mutant GRP78-GFP transfected HEK293AD cells were cultured with 10 μM Hoechst 33342 solution (Thermo-Fisher Scientific, 62249) for 1 hr at 37°C, and then imaged in phenol red-free RPMI-1640 media supplemented with 10 mM HEPES, 5 mM glutamine and 10% FBS. Live cells images were acquired with the Leica SP8 LIGHTNING Confocal Microscope with 63× oil objective.

Subcellular fractionation

H1838 cells were seeded at a density of 5×10^6 cells in a 10 cm dish 24 hr before collection in 500 μl cell fractionation buffer (20 mM HEPES, pH 7.5, 10 mM KCl, 2 mM $MgCl_2$, 2 mM EDTA, 1 mM dithiothreitol (DTT) and protease inhibitor). 100 μl was taken as whole cell lysate. The remaining cells were passed ten times through a 27-gauge needle. Nuclei and cytoplasm were separated by centrifugation at 750g for 5 min. The supernatant was taken as cytoplasmic fraction

and the nuclear pellet washed three times with cell fractionation buffer. Cold lysis buffer (0.5% NP-40, 50 mM Tris-HCl, pH 7.5, 100 mM NaCl, 0.1 mM EDTA, 10% glycerol and 1 mM dithiothreitol) was added to all fractions. Complete lysis was achieved by freezing and thawing. Lysates were spun down at 14,000g for 20 min to remove insoluble components and sodium dodecyl sulfate (SDS) loading buffer was added before Western blot analysis.

GRP78 protein refolding assay

HEK293AD cells were seeded on 6-well plate and allowed to attach overnight. Next day, the cells were transfected with Renilla Luciferase expressing construct and pcDNA3 empty vector or vectors expressing the F-78(WT), F-78(NLS Mut), or F-78(G227D). Cell lysates were collected 48 hr after transfection and subjected to heat-shock at 40°C for 1 hr. Renilla Luciferase activity was measure at 1-hr intervals for the next 4 hr. All assays were performed in the presence of an ATP-regenerating system (3 mM phosphoenol pyruvate, 20 μg/ml pyruvate kinase, 2 mM ATP). Luciferase activity was detected by a luminometer (BMG LabTech FLUOstar OPTIMA) when Luciferase Assay Buffer (Promega) was added. All experiments were performed in triplicate.

Co-immunoprecipitation

HEK293AD cells were transfected with ID2-Myc-FLAG or co-transfected with ID2-Myc-FLAG and F-78 constructs. After 24 hr of transfection, cells were lysed by sonication in IP buffer (20 mM Tris–HCl pH 7.5, 150 mM NaCl, 2 mM EDTA, 0.1% Triton X-100) supplemented with protease inhibitor cocktail (Roche). Cell lysates were centrifuged at 13,000 RPM for 30 minutes at 4°C and the clarified supernatant was saved. Immunoprecipitation was carried out by incubating the supernatants with anti-FLAG antibody or anti-Myc antibody (Thermo-Fisher Scientific, 9E10) for 1 hr at room temperature, followed by incubation with protein A agarose magnetic beads (Thermo-

Fisher Scientific) at 4°C overnight. The beads were wash three times with IP buffer, and the immunoprecipitated proteins were eluted in 2X SDS sample buffer and detected by Western blot.

Proximity ligation assay

Duolink Proximity Ligation Assay (PLA) was purchased from Millipore-Sigma (DUO92102, MilliporeSigma). HEK293AD cells were plated in a sterile Millicell EZ SLIDE 8-well glass (PEZGS0816, Millipore-Sigma) at a density of 80,000 cells per well. Following co-transfection with GRP78(WT)-GFP and ID2-FLAG or GRP78(NLS Mut)-GFP and ID2-FLAG for 48 hr, the cells were fixed with paraformaldehyde (4%, 20 min) and permeabilized in Triton X-100 (0.2% v/v, 30 min) before starting PLA. Thereafter, the PLA protocol was followed to manufacturer's specifications. Cells were imaged on Leica SP8 LIGHTNING Confocal Microscope with 63× oil objective. The primary antibodies used in PLA were rabbit anti-GFP antibody (1:100, Abcam, ab6556) and mouse anti-FLAG M2 antibody (1:200, Sigma-Aldrich, F1804).

RNA-Seq analysis

H1975 cells were seeded on 6cm dishes and allowed to attached overnight. Next day, the cells were transfected with si78(3'UTR) for 16 hr. Cells were then transfected with F-78(WT) or F-78(NLS Mut) for 48 hr. Total RNA was extracted by TRI Reagent (MilliporeSigma). RNA purity was measured by the NanoDrop 1000 (Thermo Fisher Scientific). Library preparation and RNA-Seq were performed by Novogene (Sacramento, CA) using an Illumina HiSeq 2500 System (2x150bp configuration, single index, per lane).

Bioinformatics analysis

Reads were mapped to the full human genome (GrCh38.p14) using STAR (v 2.7.9a) to generate gene counts. Counts were passed into DESeq2 (v 1.36.0) to perform differential expression analysis, removing genes with fewer than 10 counts. Heatmaps were generated using the pheatmap package (v 1.0.12). Gene ontology analysis of enriched pathways of differentially expressed genes was performed using clusterProfiler (v 4.6.0).

Cell invasion transwell assay

Transwell invasion assay was performed using the BD BioCoat Matrigel invasion chambers (BD Biosciences, #354480), according to the manufacturer's protocol. H1975 cells were seeded on 6-well plate and allowed to attached overnight. Next day, the cells were transfected with si78(3'UTR) for 16 hr followed by transfection with F-78(WT) or F-78(NLS Mut) for 24 hr. Then, transfected cells were seeded at 5,000 cells per transwell with serum-free RMPI-1640 in the upper compartment. RPMI-1640 media supplemented with 10% FBS was placed in the lower compartment of the chamber as a chemoattractant. Following a 24 hr incubation period, the non-invading cells in the upper compartment of the chamber were removed and the membranes were fixed with 4% PFA for 20 min at room temperature, washed with PBS and stained with DAPI for 30 min at room temperature. The number of cells remained on the microporous membrane or migrated to the lower compartment was counted under a confocal microscope.

Statistical analysis

All pair-wise comparisons were analyzed by GraphPad Prism v8.3.0 (GraphPad Software, San Diego, CA) using the two-tailed Student's *t* test. Data are presented as mean ± standard error of the mean (S.E.M.). A p-value of ≤ 0.05 is signified by *, p-value of ≤ 0.01 by **, p-value of ≤ 0.001 by ***, and p-value of ≤ 0.0001 by ****, n.s. denotes not significant.

Reference

1. Zhang, Y.; Liu, R.; Ni, M.; Gill, P.; Lee, A. S., Cell surface relocalization of the endoplasmic reticulum chaperone and unfolded protein response regulator GRP78/BiP. *J Biol Chem* **2010,** *285* (20), 15065-15075.

Figure S1

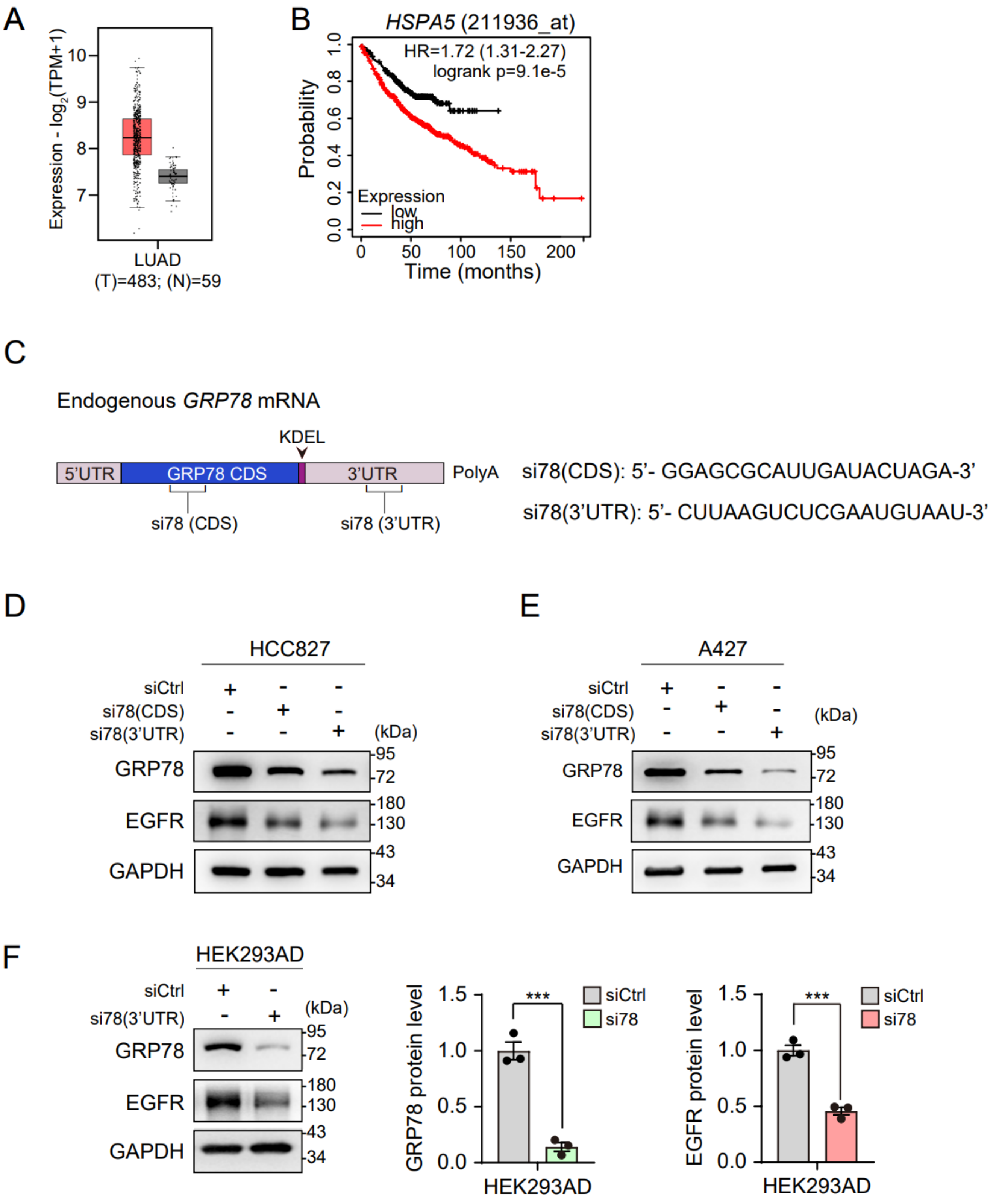

A
Expression - log2(TPM+1)
10
9
8
7
LUAD
(T)=483; (N)=59
B
HSPA5 (211936_at)
HR=1.72 (1.31-2.27)
logrank p=9.1e-5
Probability
Expression
low
high
Time (months)
C
Endogenous GRP78 mRNA
KDEL
5'UTR
GRP78 CDS
3'UTR
PolyA
si78 (CDS)
si78 (3'UTR)
si78(CDS): 5'- GGAGCGCAUUGAUACUAGA-3'
si78(3'UTR): 5'- CUUAAGUCUCGAAUGUAAU-3'
D
HCC827
siCtrl
si78(CDS)
si78(3'UTR)
(kDa)
GRP78
EGFR
GAPDH
E
A427
F
HEK293AD
GRP78 protein level
EGFR protein level
siCtrl
si78
***

Supplemental Figure S1, related to Figure 1. GRP78 expression status and survival analysis in LUAD patients and effects of GRP78 knockdown on EGFR protein levels. (A) *GRP78* mRNA expression in normal human lung tissues (n=59) and lung adenocarcinoma tissues (n=483) from TCGA database. Data analysis was performed by the Gene Expression Profiling Interactive Analysis 2 (GEPIA2) tool. (B) Kaplan-Meier survival analysis of LUAD patients with high and low GRP78 expression (n=719) by KM Plotter. (C) Schematic illustration of endogenous *GRP78* mRNA and the region targeted by the two different siRNAs against GRP78 in the coding sequence (CDS) or the 3'UTR, along with their sequences (right). (D) HCC827 or (E) A427 cells were transfected with siCtrl or si78s (CDS or 3'UTR) for 48 hr. Whole cell lysates were subjected to Western blot analysis for GRP78 and EGFR with GAPDH serving as loading control. (F) HEK293AD cells were transfected with control siRNA (siCtrl) or siRNA targeting the 3'UTR of *GRP78* mRNA (si78) for 48 hr. Whole cell lysates were subjected to Western blot analysis for GRP78 and EGFR with GAPDH serving as loading control. Quantification of the relative protein levels of GRP78 and EGFR respectively after normalization against GAPDH levels is shown in the graphs (n=3). Data are presented as mean ± S.E.M. ***p ≤ 0.001 (Student's *t* test).

Figure S2

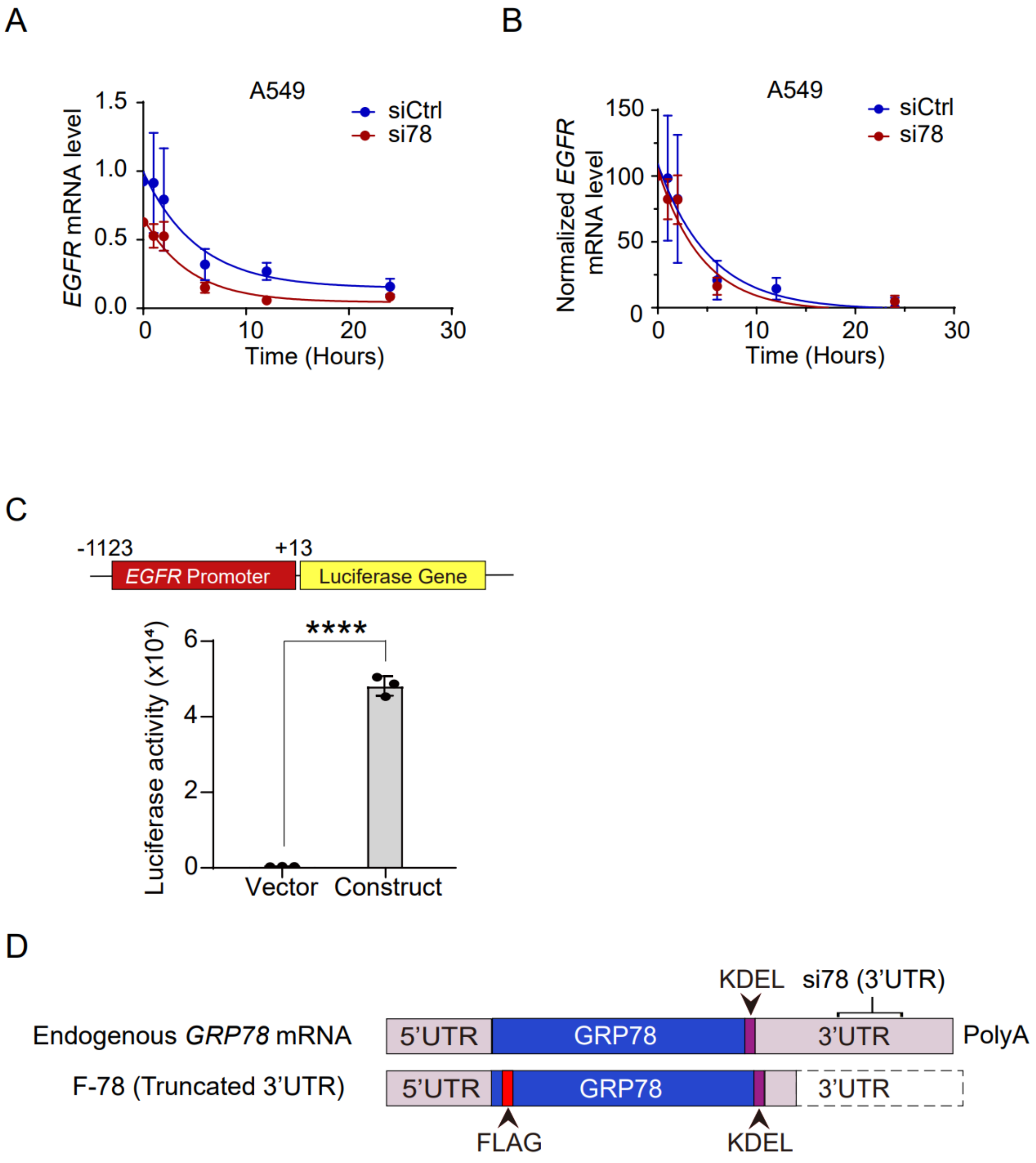
A
A549
siCtrl
si78
EGFR mRNA level
1.5
1.0
0.5
0.0
0
10
20
30
Time (Hours)
B
A549
siCtrl
si78
Normalized EGFR mRNA level
150
100
50
0
0
10
20
30
Time (Hours)
C
-1123
+13
EGFR Promoter
Luciferase Gene
****
Luciferase activity (x10⁴)
6
4
2
0
Vector
Construct
D
KDEL
si78 (3'UTR)
Endogenous GRP78 mRNA
5'UTR
GRP78
3'UTR
PolyA
F-78 (Truncated 3'UTR)
5'UTR
GRP78
3'UTR
FLAG
KDEL

Supplemental Figure S2, related to Figure 2. GRP78 knockdown does not affect *EGFR* mRNA stability and creation of EGFR-Luc reporter construct. (A) A549 cells were transfected with siCtrl or si78 for 24 hr and treated with Actinomycin D for up to 24 hr. *EGFR* mRNA levels in siCtrl or si78 treated cells were monitored at the indicated intervals by RT-qPCR and graphed (n=3). (B) Same as in (A) except initial *EGFR* mRNA level was normalized to 100%. (C) Schematic drawing of the *EGFR* promoter-driven firefly luciferase reporter construct (upper), and promoter activity assay in HEK293AD cells transfected with empty vector or the *EGFR* promoter-driven firefly luciferase reporter construct for 24 hr (lower). (D) Schematic illustrations of endogenous *GRP78* mRNA sequence with the 3' UTR region targeted by the siRNA (upper), and FLAG-tagged full-length GRP78 (F-78) (lower). Data are presented as mean ± S.E.M. ****p ≤ 0.0001 (Student's *t* test).

Figure S3

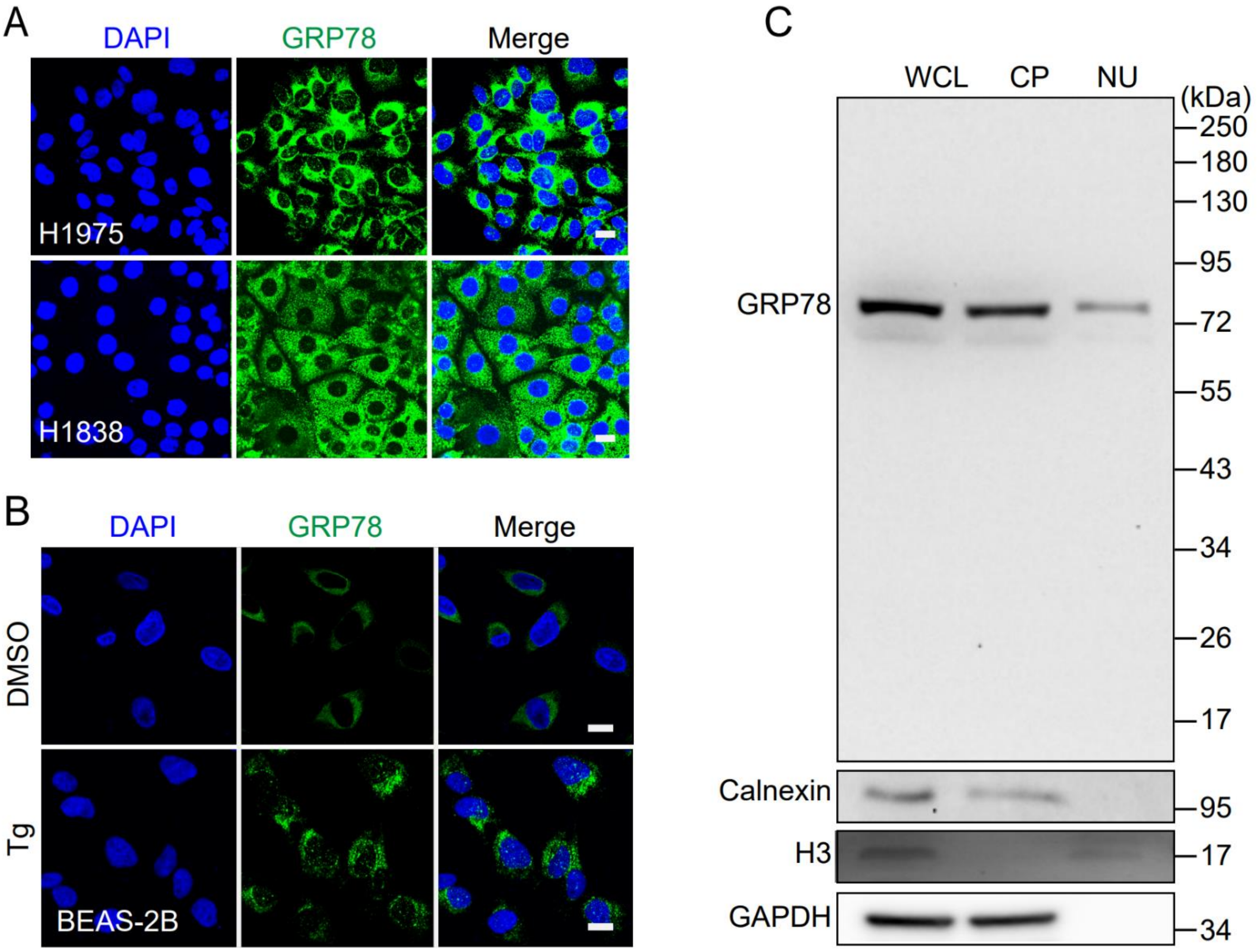

Supplemental Figure S3, related to Figure 3. GRP78 localizes to the nucleus in human lung cancer cells and cells under ER stress. (A) Representative lower magnification confocal immunofluorescence images of GRP78 (green) staining in human lung cancer H1975 (upper), and H1838 (lower) cells. DAPI (blue) represents nuclei staining. Scale bars, 10μm. (B) Representative confocal immunofluorescence lower magnification images of GRP78 (green) staining in normal human epithelial BEAS-2B cells treated with DMSO (upper) or Tg (lower) for 16 hr. DAPI (blue) represents nuclei staining. Scale bars, 10μm. (C) Western blot of whole cell

lysate (WCL), cytoplasmic (CP), and nuclear (NU) fractions of H1838 cells for GRP78 performed with a monoclonal antibody, with calnexin, Histone H3, and GAPDH serving as ER, nuclear, cytoplasmic markers respectively.

Figure S4

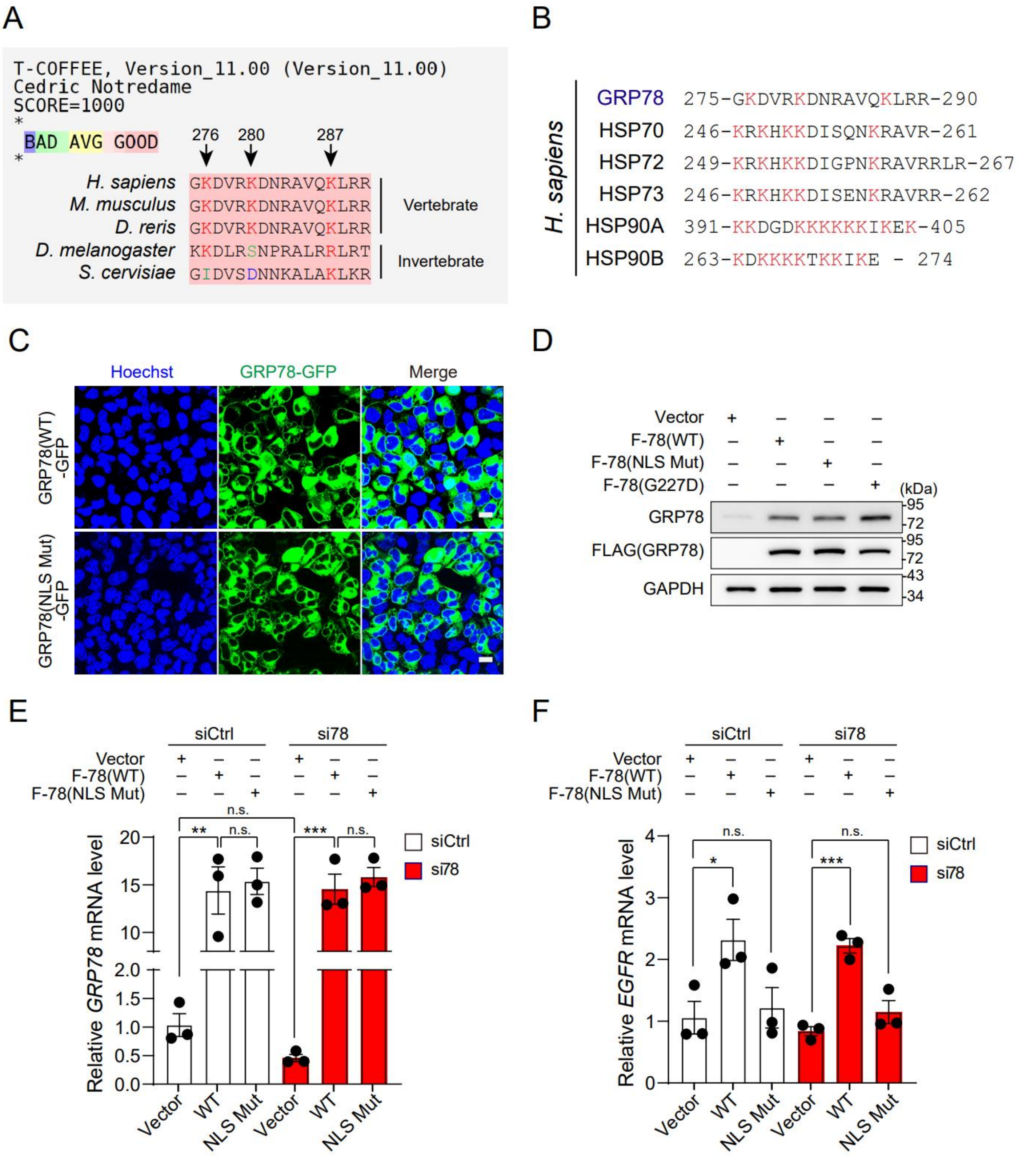

A
T-COFFEE, Version_11.00 (Version_11.00)
Cedric Notredame
SCORE=1000
BAD AVG GOOD
276 280 287
H. sapiens GKDVRKDNRAVQKLRR
M. musculus GKDVRKDNRAVQKLRR
D. reris GKDVRKDNRAVQKLRR
D. melanogaster KKDLRSNPRALRRLRT
S. cervisiae GIDVSDNNKALAKLKR
Vertebrate
Invertebrate
B
H. sapiens
GRP78 275-GKDVRKDNRAVQKLRR-290
HSP70 246-KRKHKKDISQNKRAVR-261
HSP72 249-KRKHKKDIGPNKRAVRRLR-267
HSP73 246-KRKHKKDISENKRAVRR-262
HSP90A 391-KKDGDKKKKKKIKEK-405
HSP90B 263-KDKKKKTKKIKE - 274
C
Hoechst
GRP78-GFP
Merge
GRP78(WT) -GFP
GRP78(NLS Mut) -GFP
D
Vector
F-78(WT)
F-78(NLS Mut)
F-78(G227D)
(kDa)
GRP78
FLAG(GRP78)
GAPDH
95
72
95
72
43
34
E
siCtrl
si78
Vector
F-78(WT)
F-78(NLS Mut)
n.s.
**
***
Relative GRP78 mRNA level
Vector
WT
NLS Mut
F
Relative EGFR mRNA level
*

Supplemental Figure S4, related to Figure 4. Conservation of the GRP78 nuclear localization signal among eukaryotes and other members of the chaperone protein family (A) Comparative analysis of the putative NLS sequence in GRP78 protein in different species. (B) Comparative analysis of the putative NLS sequence in different *H. sapiens* chaperone proteins. (C) Representative low magnification confocal live cell images of HEK293AD cells transfected with GRP78(WT)-GFP (upper) or GRP78(NLS Mut)-GFP constructs (lower) for 48 hr. Hoechst 33342 in blue represents nuclei staining. Scale bars, 10μm. (D) HEK293AD cells were transfected with empty vector, or F-78(WT), or F-78(NLS Mut), or F-78(G227D) for 48 hr. Whole cell lysates were subjected to Western blot analysis for GRP78 and FLAG-tagged protein with GAPDH serving as loading control. (E) RT-qPCR analysis of mRNA levels of *GRP78* and (F) *EGFR* normalized to *β-actin* in HEK293AD cells transfected with either siCtrl or si78 in combination with empty vector, F-78(WT), or F-78(NLS Mut) constructs as indicated for 24 hr. Data are presented as mean ± S.E.M. *p ≤ 0.05, **p ≤ 0.01, ***p ≤ 0.001, n.s. denotes not significant (Student's *t* test).

Figure S5

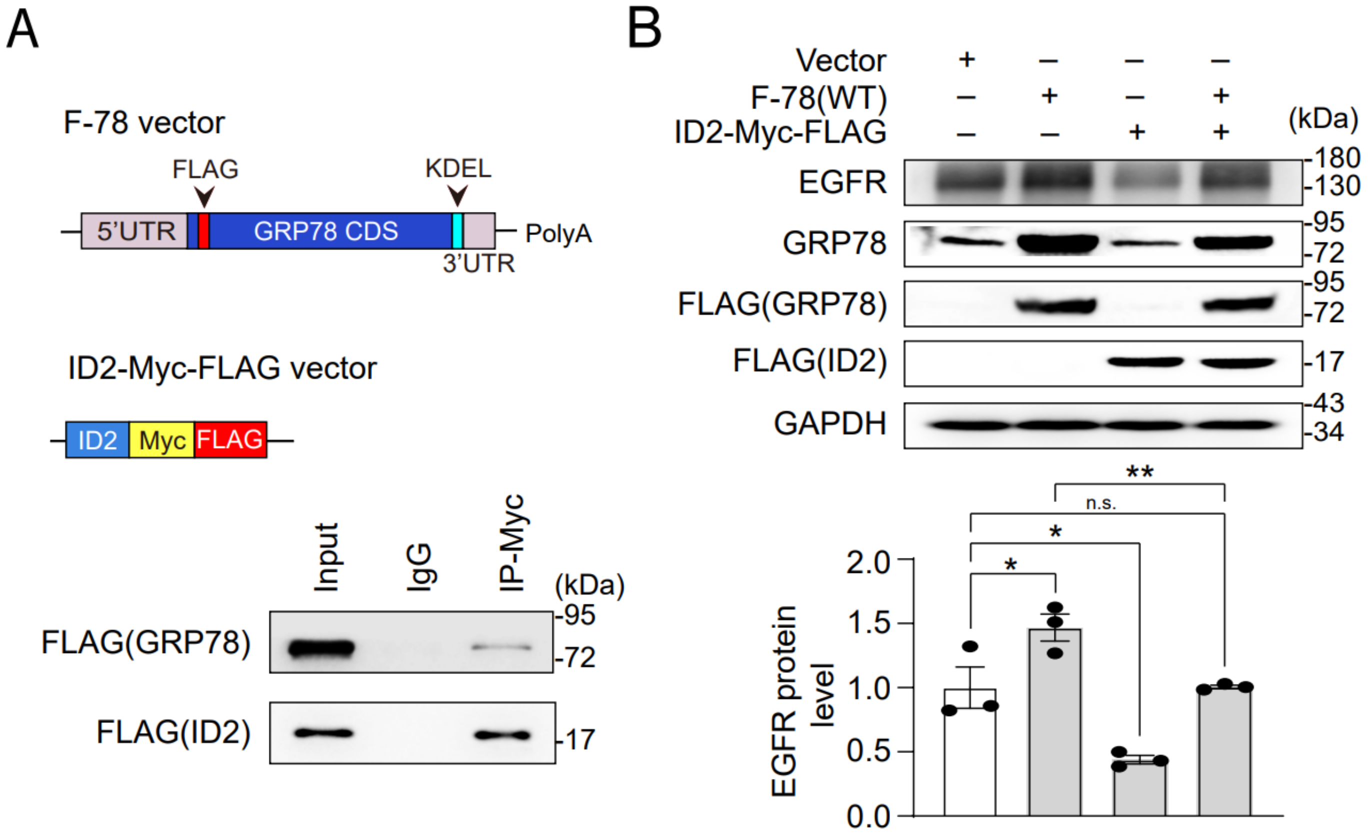

Supplemental Figure S5, related to Figure 5. Complex formation between GRP78 and ID2, a suppressor of EGFR protein expression (A) Schematic illustration of the F-78 and ID2-Myc-FLAG (upper) and immunoprecipitation assay in HEK293AD cells transfected with F-78 and ID2-Myc-FLAG constructs (lower). The immunoprecipitated proteins were probed with anti-FLAG antibody to detect GRP78 and ID2 in Western blot. (B) HEK293AD cells were transfected with empty vector or F-78(WT) or ID2-Myc-FLAG or combination of F-78(WT) and ID2-Myc-FLAG constructs as indicated for 48 hr. Whole cell lysates were subjected to Western blot for EGFR, GRP78 and FLAG-tagged proteins with GAPDH serving as loading control. Quantitation of the relative EGFR protein levels normalized against GAPDH was shown the graph below (n=3). Data are presented as mean ± S.E.M. $^{*}p \leq 0.05$, $^{**}p \leq 0.01$, n.s. denotes not significant (Student's *t* test).

Figure S6

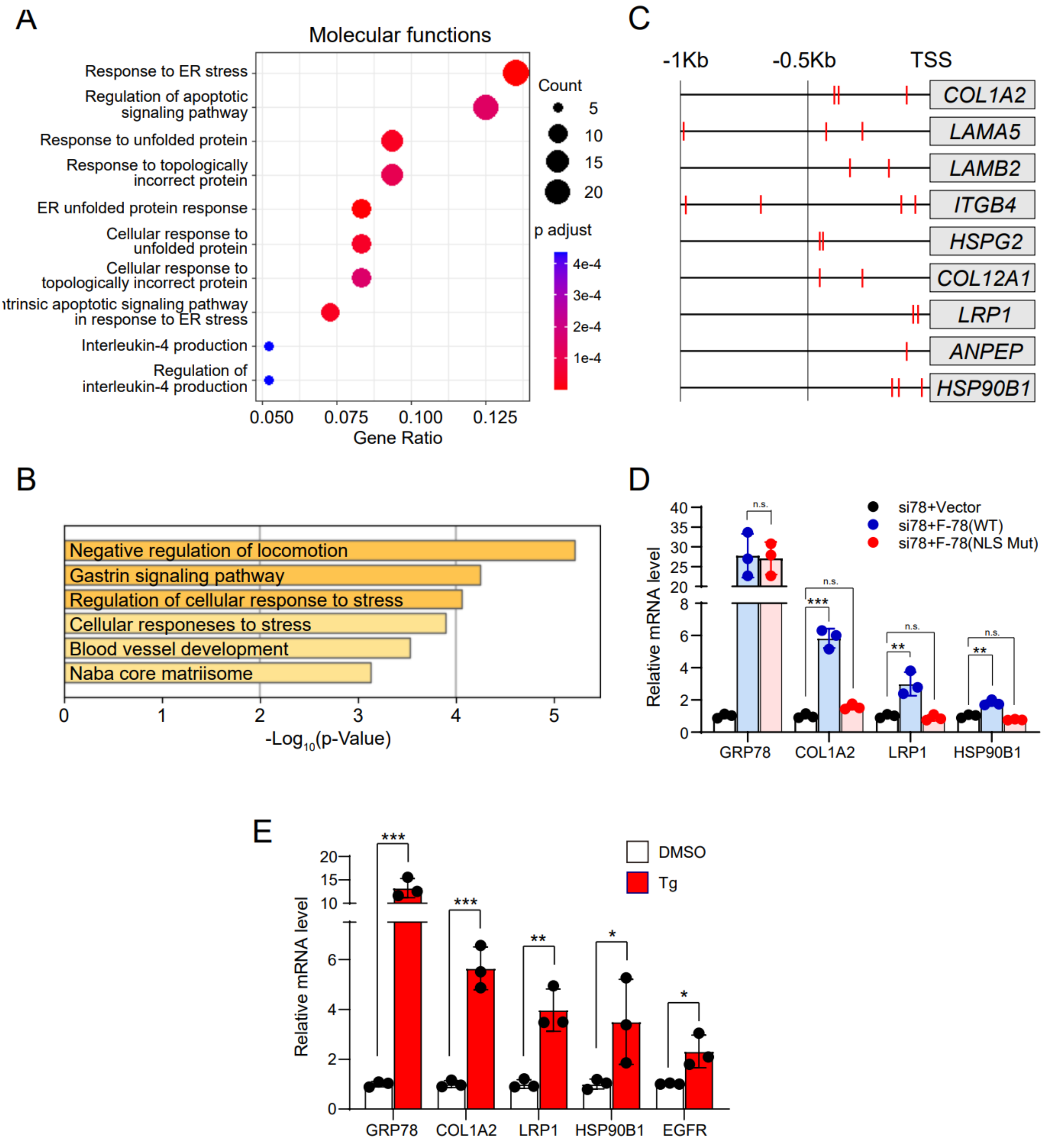

A
Molecular functions
Response to ER stress
Regulation of apoptotic signaling pathway
Response to unfolded protein
Response to topologically incorrect protein
ER unfolded protein response
Cellular response to unfolded protein
Cellular response to topologically incorrect protein
in response to ER stress
Interleukin-4 production
Regulation of interleukin-4 production
Count
5
10
15
20
p adjust
4e-4
3e-4
2e-4
1e-4
0.050
0.075
0.100
0.125
Gene Ratio
B
Negative regulation of locomotion
Gastrin signaling pathway
Regulation of cellular response to stress
Cellular responeses to stress
Blood vessel development
Naba core matriisome
0
1
2
3
4
5
-Log10(p-Value)
C
-1Kb
-0.5Kb
TSS
COL1A2
LAMA5
LAMB2
ITGB4
HSPG2
COL12A1
LRP1
ANPEP
HSP90B1
D
si78+Vector
si78+F-78(WT)
si78+F-78(NLS Mut)
Relative mRNA level
n.s.
***
**
GRP78
COL1A2
LRP1
HSP90B1
E
DMSO
Tg
Relative mRNA level
***
**
*
GRP78
COL1A2
LRP1
HSP90B1
EGFR

Supplemental Figure S6, related to Figure 6. Genes and pathways regulated by nuclear GRP78 in human lung cancer cells. (A) Pathway analysis of upregulated genes in F-78(WT) compared to empty vector control using ClusterProfiler. (B) Pathway analysis of commonly upregulated genes in both F-78(WT) and F-78(NLS Mut) compared to empty vector control in H1975 cells. (C) The location of E-Box sequence in the promoter regions of upregulated genes in F-78(WT) vs F-78(NLS Mut). (D) Validation of upregulated genes in F-78(WT) vs F-78(NLS Mut) by RT-qPCR analysis in human lung cancer H1838 cells. (E) RT-qPCR analysis of *GRP78*, *COL1A2*, *LRP1*, *HSP90B1*, and *EGFR* mRNA levels normalized to *β-actin* in BEAS-2B cells treated with DMSO or thapsigargin (Tg, 100nM) for 24 hr (n=3). Data are presented as mean ± S.E.M. *$p \le 0.05$, **$p \le 0.01$, ***$p \le 0.001$, n.s. denotes not significant (Student's *t* test).

Figure S7

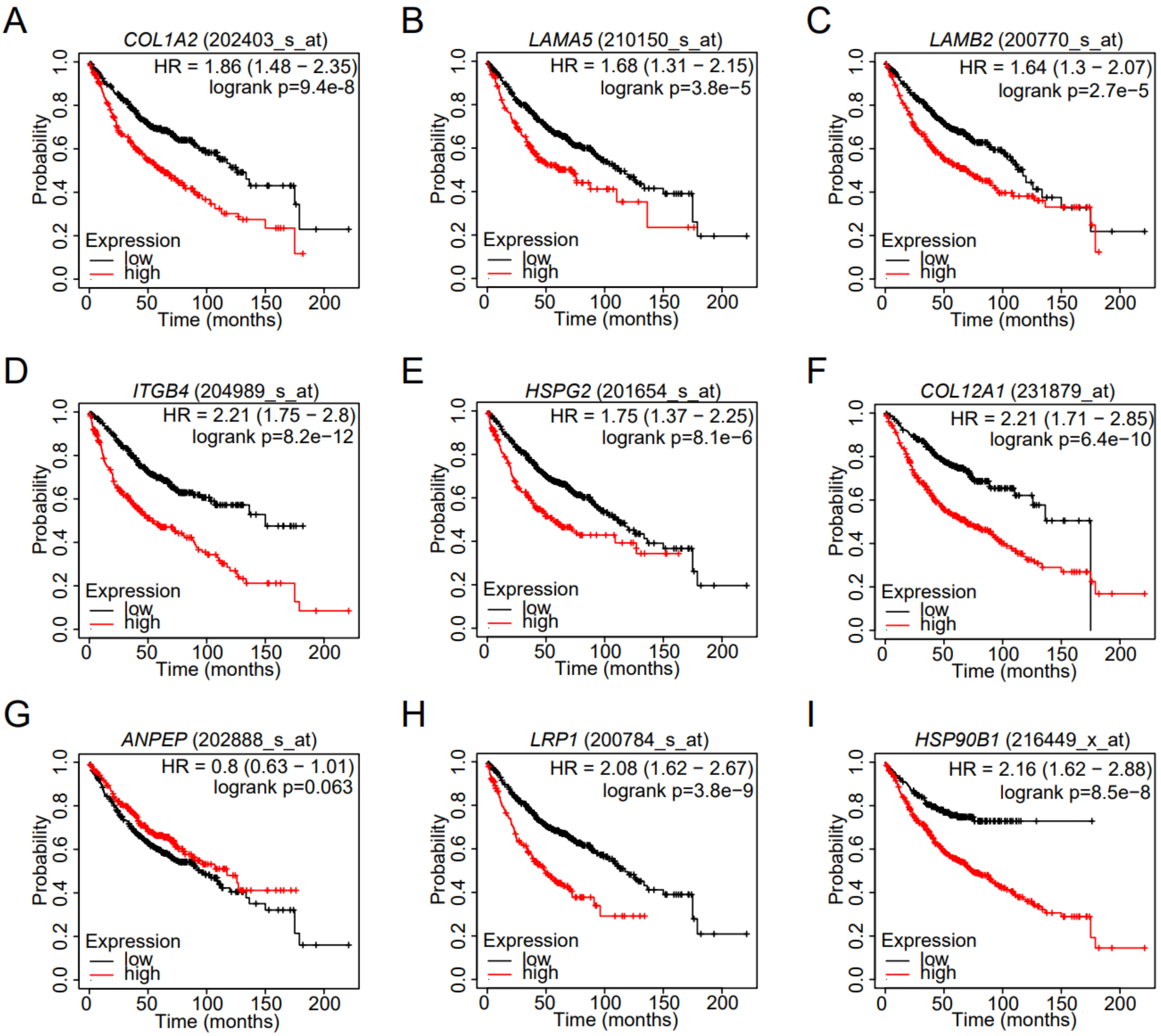

Supplemental Figure S7, related to Figure 6. Survival Analysis of the indicated upregulated genes in F-78(WT) compared to F-78(NLS Mut) in LUAD patients.

Table S1

Cell line	Histology	*EGFR* mutation	*EGFR* Amplification
H1975	Adenocarcinoma (NSCLC)	L858R/T790M	-
HCC827	Adenocarcinoma (NSCLC)	ΔE746-A750	+
H1838	Adenocarcinoma (NSCLC)	-	+
A549	Adenocarcinoma (NSCLC)	-	-
A427	Adenocarcinoma (NSCLC)	-	-
H522	Adenocarcinoma (NSCLC)	-	-
H1993	Adenocarcinoma (NSCLC)	-	-

Table S1. Mutational and amplification status of *EGFR* gene in a panel of LUAD cell lines used in the study.
